# Supplementary material for: Allosteric regulation and crystallographic fragment screening of SARS-CoV-2 NSP15 endoribonuclease
Source: Nucleic Acids Res. 2023 Apr 28;51(10):5255–70. doi: 10.1093/nar/gkad314 (PMC10250223; doi:10.1093/nar/gkad314)
Supplement: gkad314_Supplemental_Files [file gkad314_supplemental_files.zip › Supplementary datasets.zip!/224/Refine_9/01-BUSTER/Cycle-4/LIST.html]

Refining

**BUSTER 2.X**: A suite of programs for macromolecular structure refinement by maximum-likelihood and Bayesian methods  
Copyright © 1992-2020 Global Phasing Ltd.  
All rights reserved.  
Authors: G. Bricogne, E. Blanc, M. Brandl, C. Flensburg, P. Keller, W. Paciorek, P. Roversi, O. Smart, C. Vonrhein, T. Womack  

Please reference use of the software as:

- BUSTER-TNT 2.X, Global Phasing Ltd, Sheraton House, Cambridge CB3 0AX, UK

Version: 2.10.3 for linux64-ifort made on May 20 2020 at 01:14:34  
Licenced to: Diamond Light Source, Scientific Software  

```
---


### Parsing of the BUSTER cardfile

TITLE : autoBUSTER refinement

   Cell information

   Symmetry information

 Using CCP4 X-ray form factors.

   Missing structure chemical composition

 Using ATOM block for chemical composition.


   Shell information

### Parsing of the TNT cardfile


---


### Reading observations file

 Number of reflexions in input:  58343

      Number of accepted reflexions:  52749

      Number of rejected reflexions:      0

   Reflexions in test set:            2702

   Reflexions in working set:        50047


---


### 1. Generating initial model

 2.10 * Shannon sampling rate. NX NY NZ:   288  288  240


  Effective Shannon rates are:    2.29    2.29    2.55


   Allocated BUFFER of size:     76.465 Mb.

   Initial Geometry Information


 No missing atoms declared


   Summary of model volumes

 No missing atoms declared: no prior computed


 Computing bulk solvent model scattering


 NCSMASK PDB radius for solvent mask:     2.15 Angstrom

 running PDBSET (setting cell and spacegroup P1) ... done
 running NCSMASK [1] (creating mask, SMOOTH 1.04842) ... done
 running MAPDUMP (to check if reduction to unit cell is needed) ... done
 running MAPMASK (converting mask to map) ... done

   Generation of babslv by theta-filter

   Electron density in frg mask:  0.420 Å-1

 FOBS  put on rough absolute scale by Wilson plot :
           K        =  0.63883E+02
           B        =     -7.133

 

### Cycle 0001: MaxLik scaling and refinement


   MaxLik Scaling Cycle 0001

   Wilson plot

   Statistics in resolution bins just after scaling
```

|  |  |
| --- | --- |
|  | - Rxpct factors plot- Rxpct factors table- <Log-Lik Gain>- Ln Sigma A- Correlation Coefficients |

```
   <Log-Likelihood> of null-hypothesis

Structure Factor Amplitudes


   Average Figure of Merit

 Switching off refinement of overall temperature factor


 Added B =     0.3821 Å2 to all atoms in fragment


 Added B =     0.3821 Å2 to B_SOLV

```
gelly refinement module
 Authors (2004-2009) O. S. Smart, C. Flensburg, W. Paciorek, C. Vonrhein, 
                     M. Brandl, T. O. Womack and G. Bricogne
         (2010-2011) O. S. Smart, C. Flensburg, W. Paciorek, C. Vonrhein, 
                     T. O. Womack and G. Bricogne
         (2012-2014) O. S. Smart, C. Flensburg, W. Paciorek, C. Vonrhein, 
                     and G. Bricogne
         (2015-2020) G. Bricogne, C. Flensburg, W. Paciorek, C. Vonrhein
 Copyright © 2004-2020 Global Phasing Ltd. 
 All rights reserved.
 gelly module compilation date May 20 2020 01:11:20
 
 gelly uses routines from TNT geometry. References: 
     Tronrud, D. E., Ten Eyck, L. F., Matthews, B. W. (1987)
     "An Efficient General-Purpose Least-Squares Refinement Program 
     for Macromolecular Structures". Acta Crystallogr A, 43:489-501.
     Tronrud, D. E. (1997). "The TNT Refinement Package", 
     in Macromolecular Crystallography, Part B, Eds Charlie Carter 
     and Robert Sweet, Methods in Enzymology, 277:306-319.
 wildcard matching function by Julian Robichaux http://www.nsftools.com

 No -random_seed flag found. Random number stream initialised using:
2147483562
2147483398

 Control flags set to:  -autoncs -hydrogens_input_occ2zero \
                     -hydrogens_output_occ2heavy -screen \
                     100 -screen_sigma \
                     3.0 -keeppdb \
                     1000000 -taomonitor_verbose \
                     2 
 

 ############### Starting setup via TNT routines

 Setting up tnt routines including license check:

                   TNT Stereochemistry Program
 Version: 5.22.3.0
 Licence for Diamond Light Source, Scientific Software is valid
 
 Using TNT geometry routines to load the tnt control file: gelly.tnt
 Using TNT routines to load initial coordinates from file fragment.cor
 Expecting 6506 atoms

 Found     2197 "NOTE BUSTER_RESIDUE_ATOM_DATA" cards
 Have copied information for    6506 atoms from TNT to gelly's store 
 
 The TNT WEIGHT cards used by this run:
 -----WEIGHT       BOND     2.0
 -----WEIGHT       ANGLE    2.0
 -----WEIGHT       IMPROPER 0.0
 -----WEIGHT       TORSION  2.0
 -----WEIGHT       PSEUDO   0.0
 -----WEIGHT       TRIGONAL 2.0
 -----WEIGHT       PLANE    5.0
 -----WEIGHT       CONTACT  5.0
 -----WEIGHT       BCORREL  10.0
 -----WEIGHT       CHIRAL   5.0
 -----WEIGHT       NCS      50.0
 -----WEIGHT       IDEAL    4.0
 -----WEIGHT       RFACTOR  7.08
 
 Dictionary versions picked up from NOTE BUSTER_RESTRAINT_DICT cards as: 
 ------ protgeo_eh99.dat (V1.6.4.17) 20191001  STANDARD AMINO ACID DICTIONARY. BONDS AND ANGLES FROM ENGH AND HUBER EH99. OTHER VALUES BASED ON PREVIOUS TNT OR TAKEN FROM CCP4. INCLUDES HYDROGEN ATOMS.
 ------ exoticaa.dat (V1.3.4.15) 20200327  COLLECTION OF NON-STANDARD AMINO ACIDS, MAINLY EH91 WITHOUT IDEAL DISTANCE INFO
 ------ nuclgeo.dat (V1.13.4.8) 20190909
 ------ bcorrel.dat (V1.15.10.2) 20190616
 ------ contact.dat (V1.15.12.10) 20191121
 ------ idealdist_contact.dat (V1.3.4.12) 20191001  IDEAL-DISTANCE CONTACT TERM DATA AS USED IN PROLSQ. VALUES USED HERE ARE BASED ON DATA HARVESTING OF PDB AS OF 20190109.
 ------ restraints for LIG (Unknown) from cif dictionary Z927400026.cif
 ------ restraints for CIT (CITRIC ACID) from cif dictionary CIT.cif; generated by GRADE_PDB_LIGAND 1.2.12 (October 4 2016) using MOGUL 1.7.2(RC1), CSD as538be, with quantum mechanics RM1 ((DatabaseID PDB-CIT))
 ------ assume.dat (V1.9.4.2) 20180705
 
 Have picked up spacegroup name for pdb output as 'P 63      '
 Setting up Chain/Residue arrays
 
 Have command line specification -autoncs*
     Have found       1 NCS relations
     Pairwise matching residue number and type between chains: 
                    A        B        S        W    
          A         .       99%       -        -   
          B                  .        -        -   
          S                           .        -   
          W                                    .   
       Table key--                                                       
          all   : two chains have 100% match and same number of residues 
         multi  : ditto and they contribute to a multi chain ncs cluster 
          ***%  : two chains have >80% matching residue number/type      
           -    : chains appear to be unrelated                          
      chain A     have identified ncs to     1 other chains. So will weight each autoncs by (       6.666/#)=      6.6660
      chain B     have identified ncs to     1 other chains. So will weight each autoncs by (       6.666/#)=      6.6660
      chain S     have identified ncs to     0 other chains. 
      chain W     have identified ncs to     0 other chains. 
     For .Gelly cards produced see below - after pruning
 
 Setting up BUSTER_SET groups
 Have found    89 BUSTER_SET cards
 Now silently applying automatic/dictionary set definitions. For a verbose listing use '-verbose_set' flag.
 Applying user defined set definitions:
 =====NOTE BUSTER_SET ncsautoXcld = Water --> set 'ncsautoXcld' now has   966 atoms 
 =====NOTE BUSTER_SET ncsautoABset = Chain_A \ ncsautoXcld --> set 'ncsautoABset' now has  2770 atoms 
 Have defined    49 sets: 
      set:                 'All' has   6506 atoms in   1666 residues - automatically defined
      set:               'Empty' has      0 atoms in      0 residues - automatically defined
      set:            'ExoticAA' has      0 atoms in      0 residues - defined by dictionary file
      set:           'ExoticNUC' has      0 atoms in      0 residues - defined by dictionary file
      set:              'NotHET' has   5514 atoms in    698 residues - defined by dictionary file
      set:             'Chain_A' has   2770 atoms in    350 residues - automatically defined
      set:             'Chain_B' has   2770 atoms in    350 residues - automatically defined
      set:             'Chain_S' has    567 atoms in    567 residues - automatically defined
      set:             'Chain_W' has    399 atoms in    399 residues - automatically defined
      set:               'Water' has    966 atoms in    966 residues - defined by dictionary file
      set:          'StdProtein' has   5514 atoms in    698 residues - defined by dictionary file
      set:             'Protein' has   5514 atoms in    698 residues - defined by dictionary file
      set:                'Back' has   2794 atoms in    698 residues - defined by dictionary file
      set:                'Side' has   2720 atoms in    650 residues - defined by dictionary file
      set:        'RnaCanonical' has      0 atoms in      0 residues - defined by dictionary file
      set:        'DnaCanonical' has      0 atoms in      0 residues - defined by dictionary file
      set:     'RnaDnaCanonical' has      0 atoms in      0 residues - defined by dictionary file
      set:     'DnaRnaCanonical' has      0 atoms in      0 residues - defined by dictionary file
      set:         'RnaExtended' has      0 atoms in      0 residues - defined by dictionary file
      set:         'DnaExtended' has      0 atoms in      0 residues - defined by dictionary file
      set:      'RnaDnaExtended' has      0 atoms in      0 residues - defined by dictionary file
      set:      'DnaRnaExtended' has      0 atoms in      0 residues - defined by dictionary file
      set:                 'Dna' has      0 atoms in      0 residues - defined by dictionary file
      set:                 'Rna' has      0 atoms in      0 residues - defined by dictionary file
      set:              'RnaDna' has      0 atoms in      0 residues - defined by dictionary file
      set:              'DnaRna' has      0 atoms in      0 residues - defined by dictionary file
      set:             'Nucleic' has      0 atoms in      0 residues - defined by dictionary file
      set:             'Polymer' has   5514 atoms in    698 residues - defined by dictionary file
      set: 'BufferWeichenberger' has     13 atoms in      1 residues - defined by dictionary file
      set:    'BufferAdditional' has      0 atoms in      0 residues - defined by dictionary file
      set:              'Buffer' has     13 atoms in      1 residues - defined by dictionary file
      set:         'MetalAlkali' has      0 atoms in      0 residues - defined by dictionary file
      set:  'MetalAlkalineEarth' has      0 atoms in      0 residues - defined by dictionary file
      set:     'MetalTransition' has      0 atoms in      0 residues - defined by dictionary file
      set: 'MetalPostTransition' has      0 atoms in      0 residues - defined by dictionary file
      set:    'MetalLanthanides' has      0 atoms in      0 residues - defined by dictionary file
      set:               'Metal' has      0 atoms in      0 residues - defined by dictionary file
      set:         'SugarAlphaD' has      0 atoms in      0 residues - defined by dictionary file
      set:         'SugarAlphaL' has      0 atoms in      0 residues - defined by dictionary file
      set:               'Sugar' has      0 atoms in      0 residues - defined by dictionary file
      set:               'Other' has     26 atoms in      2 residues - defined by dictionary file
      set:            'Hydrogen' has      0 atoms in      0 residues - defined by dictionary file
      set:         'nonHydrogen' has   6506 atoms in   1666 residues - defined by dictionary file
      set:             'OccZero' has      0 atoms in      0 residues - defined by dictionary file
      set:          'OccNonZero' has   6506 atoms in   1666 residues - defined by dictionary file
      set:             'OccFull' has   6461 atoms in   1663 residues - defined by dictionary file
      set:          'OccNonFull' has     45 atoms in      3 residues - defined by dictionary file
      set:         'ncsautoXcld' has    966 atoms in    966 residues - user specified
      set:        'ncsautoABset' has   2770 atoms in    350 residues - user specified
 No command line arguments '-occ_allhet' or '-occ_restyp' so will not setup automated occcupancy refinement
 
 Interpreting CONSTANT and FREE cards: 
 =====CONSTANT     OCC
 Have found and interpreted      1 CONSTANT cards in the TNT input. 
 Before any COMBINE - Number of atoms with fixed XYZ =     0
 Before any COMBINE - Number of atoms with fixed B   =     0
 Before any COMBINE - Number of atoms with fixed OCC =  6506
 Before any COMBINE - Number of atoms with fixed BIJ =   966
                            cf total number of atoms =  6506
 
 There are no COMBINE cards to be found in the TNT card input 
 
 There are no NOTE BUSTER_COMBINE_RES cards to be found in the TNT card input 

 ############### Working out full connectivity list


 ############### Working out equivalent atoms


 Residue with most atoms given below:
        A|58:N(TRP)         
        A|58:CA(TRP)        
        A|58:CB(TRP)        
        A|58:CG(TRP)        
        A|58:CD1(TRP)       
        A|58:NE1(TRP)       
        A|58:CE2(TRP)       
        A|58:CD2(TRP)       
        A|58:CE3(TRP)       
        A|58:CZ3(TRP)       
        A|58:CH2(TRP)       
        A|58:CZ2(TRP)       
        A|58:C(TRP)         
        A|58:O(TRP)         


 Have no '-equiv_check_plus' argument - so will take atom type into
 account when checking for equivalence of otherwise identical atoms.
 

 ############### Looking at dihedrals


 ############### Looking at chirals

 NOTE BUSTER_CHIRAL_ANGLECUT card found - read value=    -31.00 degs from it
 NOTE BUSTER_CHIRAL_SIGMA card found - read value=      1.70 degs from it
 RANGE allowed for B variables in refinement:   from      3.00000 to    300.00000

 ############### Preparing for PDB output


 ############### Processing extra restraints explicitly defined

 No 'NOTE BUSTER_TARGET' cards or -target?? args found (used to read coordinates files for soft ncs to external)
 No 'NOTE BUSTER_OCCSUM'     cards found - so not using sum of occupancy restraints
 No 'NOTE BUSTER_DISTANCE'   cards found - so not setting up any additional atom-atom distance restraints
 No 'NOTE BUSTER_UTILANGLE'  cards found - so not setting up any additional restraints on any angle
 No 'NOTE BUSTER_UTILTOR'    cards found - so not setting up any additional sinusoidal restraints on torsions
 
 --- Have some similarity definitions 
     Similarity restraints on RMSD use superposition routines from: 
     Coutsias, E.A., Seok, C., Dill, K.A.(2004)
     "Using quaternions to calculate RMSD", J. Comput. Chem., 25:1849-1857 

 Have found    1 'NOTE BUSTER_SIM_DEFINE' cards 
     Maximum number of chains involved in any SIM group (excluding the template)=     1


 ########## Interpreting card: NOTE BUSTER_SIM_DEFINE ncsautoAB ncsautoABset B
     Number of atoms in template set=  2770
     Number of related chains (other than template) in SIM set=    1
     List of chains to be restrained (excluding template)=   B   
     Pairwise RMSD, XYZ in Angstroms, for SIM group: ncsautoAB
                 A         B   
     A         -----     ----- 
     B         0.541     ----- 
     Overall RMSD,XYZ for the SIM group is     0.541 Angstroms
     Pairwise rms deviation of [individual B's minus their set/chain average] in Angs**2
                 A         B         Bavg
     A         -----     -----     84.16
     B         22.28     -----     65.26
     Overall RMSD,B   for the SIM group is    22.276 Angs**2
     The number of common atoms for the above fits:
                 A         B   
     A         -----     ----- 
     B          2757     ----- 
     (compared to the template set that has    2770 atoms in it)
     List of atoms that are absent in one or more chains for this group: 
                                    A    B   
                    C1      401     +    -
                    O1      401     +    -
                    O2      401     +    -
                    C2      401     +    -
                    C3      401     +    -
                    O7      401     +    -
                    C4      401     +    -
                    C5      401     +    -
                    O3      401     +    -
                    O4      401     +    -
                    C6      401     +    -
                    O5      401     +    -
                    O6      401     +    -
     (key  +  atom present for this chain 
           -  atom absent  for this chain)
 transforms for each of the superpositions orthogonal matrix r11 r12 r13 r21 r22 r23 r31 r32 r33 tx ty tz (PDBSET syntax): 
TRANS_AB -0.877018 -0.480296 -0.012485 -0.480318  0.877093 -0.001365  0.011606  0.004800 -0.999921    33.884    32.998    15.300
 reverse transforms 
TRANS_BA -0.877018 -0.480318  0.011606 -0.480296  0.877093  0.004800 -0.012485 -0.001365 -0.999921    45.388   -12.741    15.767
 
 Setting up LSSR interatomic distance restraints
 Interpreting card: NOTE BUSTER_SIM_RESTRAIN_LSSR ncsautoAB 6.666
        setting up interatomic distance pair restraints with:
        Dmin=         0.00
        Dmax=         5.50
        sigma=        0.20
        Vmax=         3.00
        weight=       6.67
        number of distance pairs defined for this set is      33972
 Have not supplied either '-sim_swap_equiv' or '-sim_swap_equiv_plus' argument 
   so will not try swaping equivalent atoms to improve NCS and/or target agreement.
 
 NCS by LSSR outlier analysis
 -autoncs option automatic NCS prune routine -------- 
 Residues pruned out by gradient condition:
          A|135     A|147     A|148     A|149     A|150     A|151     A|172
          A|173     A|175     A|181     A|182     A|185     A|186     A|187
          A|188     A|189     A|216     A|217     A|218     A|219     A|220
          A|221     A|253     A|259     A|310     A|319     A|326     A|344
          A|345     A|346     B|135     B|147     B|148     B|149     B|150
          B|151     B|172     B|173     B|175     B|181     B|182     B|185
          B|186     B|187     B|188     B|189     B|216     B|217     B|218
          B|219     B|220     B|253     B|259     B|310     B|319     B|326
          B|344     B|345     B|346
  
 Residues pruned out by function/max condition:
          A|149!    A|173!    A|187!    A|188!    A|346!   A|ENDA     B|149!
          B|173!    B|187!    B|188!    B|346!   B|ENDA 
  
 Total number of residues for which LSSR restraints have been pruned out is       61 out of      698 participating
 Number of LSSR restraints pruned out is      3927 this is   11.6% of the total           33972
  
 -autoncs .Gelly cards suitable for adaptation for manual definition/pruning
           (note auto pruning is done in a slightly different way so exact results may differ slightly)
 -autoncs option .Gelly cards also written to file  auto_gelly_cards.txt
NOTE BUSTER_SET ncsautoXcld = Water    # card added - produced by -autoncs
NOTE BUSTER_SET ncsautoXcld = ncsautoXcld + { A|135 A|147 A|148 A|149 A|150 }    # card added - produced by -autoncs
NOTE BUSTER_SET ncsautoXcld = ncsautoXcld + { A|151 A|172 A|173 A|175 A|181 }    # card added - produced by -autoncs
NOTE BUSTER_SET ncsautoXcld = ncsautoXcld + { A|182 A|185 A|186 A|187 A|188 }    # card added - produced by -autoncs
NOTE BUSTER_SET ncsautoXcld = ncsautoXcld + { A|189 A|216 A|217 A|218 A|219 }    # card added - produced by -autoncs
NOTE BUSTER_SET ncsautoXcld = ncsautoXcld + { A|220 A|221 A|253 A|259 A|310 }    # card added - produced by -autoncs
NOTE BUSTER_SET ncsautoXcld = ncsautoXcld + { A|319 A|326 A|344 A|345 A|346 }    # card added - produced by -autoncs
NOTE BUSTER_SET ncsautoXcld = ncsautoXcld + { A|ENDA }    # card added - produced by -autoncs
NOTE BUSTER_SET ncsautoABset = Chain_A \ ncsautoXcld    # card added - produced by -autoncs
NOTE BUSTER_SIM_DEFINE ncsautoAB ncsautoABset B    # card added - produced by -autoncs
NOTE BUSTER_SIM_RESTRAIN_LSSR ncsautoAB 6.666    # card added - produced by -autoncs

 LSSR function contribution before pruning     26512.3
                             after pruning      9022.2
 Graph of which residues pruned from LSSR: ncs_pruned.mtv
 Graph of LSSR function outliers ncs_function_outliers.mtv
 Graph of LSSR gradient outliers ncs_gradient_outliers.mtv
 LSSR - analysis of distance difference distribution for NCS
         DeltaD less than 0.5 Angs:  proportion of population= 98.73% average DeltaD of subset     0.007 Angs
 Histogram of LSSR deltaD distribution ncs_histogram.mtv
 
 Interpreting card: NOTE BUSTER_TLS_SET tls1 { A|* }
 Interpreting card: NOTE BUSTER_TLS_SET tls2 { B|* }
 Interpreting card: NOTE BUSTER_TLS_SET tls1 { A|* }
 Interpreting card: NOTE BUSTER_TLS_T   tls1     -1.00387E-01 -1.94646E-02 -1.04862E-01 -2.75119E-02  4.61084E-02  5.22733E-03
 Interpreting card: NOTE BUSTER_TLS_L   tls1      1.43977E+00  1.66678E+00  8.28191E-01  2.91853E-01 -4.93436E-01  3.60273E-01
 Interpreting card: NOTE BUSTER_TLS_S   tls1      2.45347E-01 -2.02592E-01  2.44710E-01 -1.91009E-01 -7.07893E-02 -2.32867E-01 -5.33582E-02 -7.54950E-02
 Interpreting card: NOTE BUSTER_TLS_O   tls1     -2.34016E+01 -8.37990E+01  1.17779E+01
 Interpreting card: NOTE BUSTER_TLS_SET tls2 { B|* }
 Interpreting card: NOTE BUSTER_TLS_T   tls2     -1.34545E-01 -7.70270E-02  4.06613E-02  2.21513E-02  4.79797E-02 -1.95693E-02
 Interpreting card: NOTE BUSTER_TLS_L   tls2      1.12658E+00  1.16836E+00  9.36760E-01  5.85777E-01 -6.91255E-01 -5.92143E-01
 Interpreting card: NOTE BUSTER_TLS_S   tls2     -4.81861E-02  5.31306E-02 -8.29736E-02  4.54205E-02  2.18779E-01  5.84247E-02 -8.79871E-02  4.73445E-02
 Interpreting card: NOTE BUSTER_TLS_O   tls2      2.21707E+01 -7.20650E+01  3.37962E+00
 Interpreting card: NOTE BUSTER_TLS_FIX tls1 ALL
 Interpreting card: NOTE BUSTER_TLS_FIX tls2 ALL


 TLS information. Total number of groups:     2


 Group   Natoms    FixRB   FixTLS   Group Tag    Group Spec
     1     2770      Y        Y     tls1    { A|* }
     2     2770      Y        Y     tls2    { B|* }
 
NOTE BUSTER_TLS_SET tls1 { A|* }
NOTE BUSTER_TLS_T   tls1     -1.00387E-01 -1.94646E-02 -1.04862E-01 -2.75119E-02  4.61084E-02  5.22733E-03
NOTE BUSTER_TLS_L   tls1      1.43977E+00  1.66678E+00  8.28191E-01  2.91853E-01 -4.93436E-01  3.60273E-01
NOTE BUSTER_TLS_S   tls1      2.45347E-01 -2.02592E-01  2.44710E-01 -1.91009E-01 -7.07893E-02 -2.32867E-01 -5.33582E-02 -7.54950E-02
NOTE BUSTER_TLS_O   tls1     -2.34016E+01 -8.37990E+01  1.17779E+01
NOTE BUSTER_TLS_FIX tls1 ALL
 
NOTE BUSTER_TLS_SET tls2 { B|* }
NOTE BUSTER_TLS_T   tls2     -1.34545E-01 -7.70270E-02  4.06613E-02  2.21513E-02  4.79797E-02 -1.95693E-02
NOTE BUSTER_TLS_L   tls2      1.12658E+00  1.16836E+00  9.36760E-01  5.85777E-01 -6.91255E-01 -5.92143E-01
NOTE BUSTER_TLS_S   tls2     -4.81861E-02  5.31306E-02 -8.29736E-02  4.54205E-02  2.18779E-01  5.84247E-02 -8.79871E-02  4.73445E-02
NOTE BUSTER_TLS_O   tls2      2.21707E+01 -7.20650E+01  3.37962E+00
NOTE BUSTER_TLS_FIX tls2 ALL


 TLS: mmCIF group specification begin


#
loop_
_pdbx_refine_tls_group.id
_pdbx_refine_tls_group.refine_tls_id
_pdbx_refine_tls_group.beg_PDB_ins_code
_pdbx_refine_tls_group.beg_auth_asym_id
_pdbx_refine_tls_group.beg_auth_seq_id
_pdbx_refine_tls_group.end_PDB_ins_code
_pdbx_refine_tls_group.end_auth_asym_id
_pdbx_refine_tls_group.end_auth_seq_id
_pdbx_refine_tls_group.selection
_pdbx_refine_tls_group.selection_details
   1   1 . A   -1 . A  346 . '{ A|* }'
   2   1 . A  401 . A  401 . '{ A|* }'
   3   2 . B   -1 . B  346 . '{ B|* }'
   4   2 . B  401 . B  401 . '{ B|* }'
#


 TLS: mmCIF group specification end


 Interpreting card: NOTE BUSTER_ANISO_SET Empty
 Interpreting card: NOTE BUSTER_ANISO_WEIGHT SCA  16.0
 Interpreting card: NOTE BUSTER_ANISO_WEIGHT SPH   0.6    0.6
 Interpreting card: NOTE BUSTER_ANISO_WEIGHT SIM   7.8    4.8    2.6    0
 Interpreting card: NOTE BUSTER_ANISO_WEIGHT RIG  21.5   21.5    3.7    0


 After TLS and ANISO: Number of atoms with fixed/combined xyz =     0
 After TLS and ANISO: Number of atoms with fixed/combined B   =     0
 After TLS and ANISO: Number of atoms with fixed/combined OCC =  6506
 After TLS and ANISO: Number of atoms with fixed/combined BIJ =  6506
 After TLS and ANISO: Number of atoms in a TLS group          =  5540
                                     cf total number of atoms =  6506
 

 ############### Setup state vector

 No free_vect_ulim_xyz argument found: using default of  100.000
 No free_vect_llim_xyz argument found: using default of -100.000
 Number of parameters added for TLS description                 =       0
 Number of parameters to describe molecular conformation        =   26024

 ############### Setup free vector

 Number of free parameters to be adjusted in refinement   =   26024

 ############### Setting up own geometry


 No command line argument '-tnt_fn' so will use gelly to do geometry function calculations

 ============= Call TNT functions


 ============= Get TNT symmetry information

 
      Getting symmetry operators from TNT. 
          gelly will classify symmetry using pdb-like convention: 
               SYMOP   SYMMETRY
              NNNMMM   OPERATOR
                1555   X,Y,Z
                2555   -Y,X-Y,Z
                3555   -X+Y,-X,Z
                4555   -X,-Y,1/2+Z
                5555   Y,-X+Y,1/2+Z
                6555   X-Y,X,1/2+Z
          where NNN -> operator number and MMM -> translation vector
 

 ============= Own Gelly geometry/restraints setup


 ----------- Identify bond-types


    Setting up GEOMBONDREC_TYPE_HASH* to be able to map a given bond type (e.g.
    "single") to an internal integer (GBOND_TYPE=1) etc.

    Using default  GEOMBONDREC_TYPE_HASH_STR='SING* 1 DOUB* 2 TRIP* 3 AROM* 4 DELO* 4 METAL* 5':
    to override default use a NOTE BUSTER_GEOMBONDREC_TYPE_HASH_STR gelly card.

 ----------- Classify torsion into peptide omega and other


 ----------- Setup of non-bonded terms

 Ideal-distance contact term setup:
    Restraint function constants (normally these values are from NOTE BUSTER_IDEAL_CONTACT_* cards)
    Sigma for general contacts      0.200 Angs
    Sigma for 1-4 contacts          0.100 Angs (contacts across a torsion)
    Sigma for 1-5 contacts          0.150 Angs
    Sigma for 1-N contacts          0.200 Angs
    Sigma for HBOND pairs           0.100 Angs (contacts between HB_types A and D or B's)
    Contact distance increment for each atom in HBOND          -0.150 Angs 
    Contact distance increment for H-bond acceptor              0.100 Angs 
    Contact distance increment for 1-4 with an O atom          -0.050 Angs 
    Contact distance increment for 1-4 with an N atom          -0.180 Angs 
    Contact distance increment for 1-4 with an C atom          -0.370 Angs 
    Contact distance increment for 1-4 with an S atom          -0.150 Angs 
    Contact distance increment for 1-4 with an P atom          -0.150 Angs 
    Contact distance increment for 1-4 with any other atom     -0.150 Angs 
    All atoms matched for CCP4 atom type.
    Number of CCP4 atom types used is         25 (H-bound and not H-bound counted separately)

 Setting up short/ideal-distance contacts
   Using gelly special position handling. (For TNT instead rerun with argument '-special_tnt')

   No command line argument '-special_all' specified. We will auto-generate
   EXCLUDE cards only for atom-atom contacts across symmetry (i.e. on special
   position) when this involves a contact within a residue to itself.

   Have set up non-bonded exclusions - 1-3 contacts
   Atom A|27:CA         makes the most 1-3 contacts:  6

   Have set up non-bonded exclusions - 1-4 contacts
   Atom A|27:N          makes the most 1-4 contacts:  7

   Have set up non-bonded exclusions - 1-5 contacts
   Atom A|28:C.A        makes the most 1-5 contacts: 10

 ############################################################################################################
 ###################################################### setting up contact terms
 ############################################################################################################


 ============= Call Gelly versions of TNT functions

 WARNING: 1-4 contact without 1-(2,3)-4 data for A|401:O2(CIT)        and A|401:C3(CIT)        X-X
 WARNING: 1-4 contact without 1-(2,3)-4 data for A|401:C2(CIT)        and A|401:O6(CIT)        X-X
 WARNING: 1-4 contact without 1-(2,3)-4 data for A|401:O7(CIT)        and A|401:O5(CIT)        X-X
 WARNING: 1-4 contact without 1-(2,3)-4 data for A|401:C4(CIT)        and A|401:O6(CIT)        X-X
 WARNING: 1-4 contact without 1-(2,3)-4 data for B|401:C01(LIG)       and B|401:N05(LIG)       X-X
 WARNING: 1-4 contact without 1-(2,3)-4 data for B|401:C04(LIG)       and B|401:C13(LIG)       X-X
 WARNING: 1-4 contact without 1-(2,3)-4 data for B|401:C09(LIG)       and B|401:C12(LIG)       X-X
 WARNING: 1-4 contact without 1-(2,3)-4 data for B|401:C10(LIG)       and B|401:C13(LIG)       X-X

       TERM      weight      number      TNT_fn    Gelly_fn        diff
       BOND       2.000        5655     639.511     639.511       0.000
       ANGL       2.000        7674    3584.501    3584.501       0.000
       IMPR       0.000           0       0.000       0.000       0.000
       TORS       2.000        1968    2746.563    2746.563      -0.000
       TRIG       2.000           0       0.000       0.000       0.000
       PLAN       5.000         963    1473.801    1473.801       0.000
       CONT       5.000           0      61.992       0.000      61.992
       BCOR      10.000        5630    1696.826    4114.208   -2417.383


 ============= Call Gelly geometry functions

   Switching torsion functional form to sinusoidal rather than split harmonic functional.
      if you want this not to happen then rerun with '-torsharm'
      Weight for sinusoidal torsion=     2.000
   BCOR function/gradient will be split between H and non-H atoms (as for any atom pair). Use '-bcorrel_all_in_hydrogens' to change this.

 ----------- Fix B-correlation restraints

 
 Checking for bonds that do not have a bcorrel restraint defined
 We will wipe all existing BCORrels if OCC=0.0 atom(s) is/are involved. This can be' switched off via the '-bcorrel_nowipe_zeroocc' flag.
 We will wipe all existing BCORrels if only non-hydrogen atom(s) with fixed B(iso) are involved. This can be switched off via the '-bcorrel_nowipe_fixb_nonhydrogens' flag.
 We will keep all existing BCORrels if hydrogen atom(s) with fixed B(iso) is/are involved. This can be switched off via the '-bcorrel_wipe_fixb_hydrogens' flag.
 No -bcorrel_missing_sigma found. So will set sigma to     2.5 Ang**2 for any bond with no existing BCORrel
 No -bcorrel_missing_sigma_hydrogens found. So will set sigma to     1.0 Ang**2 for any bond with no existing BCORrel involving hydrogens

 No active BCORels found involving OCC=0.0 atom(s)


 No active BCORels found involving fixed-B (only) non-hydrogen atom(s)

    add a default BCOR for bond A|401:C1(CIT) A|401:C2(CIT)
    add a default BCOR for bond A|401:C1(CIT) A|401:O1(CIT)
    add a default BCOR for bond A|401:C1(CIT) A|401:O2(CIT)
    add a default BCOR for bond A|401:C2(CIT) A|401:C3(CIT)
    add a default BCOR for bond A|401:C3(CIT) A|401:C4(CIT)
    add a default BCOR for bond A|401:C3(CIT) A|401:C6(CIT)
    add a default BCOR for bond A|401:C3(CIT) A|401:O7(CIT)
    add a default BCOR for bond A|401:C4(CIT) A|401:C5(CIT)
    add a default BCOR for bond A|401:C5(CIT) A|401:O3(CIT)
    add a default BCOR for bond A|401:C5(CIT) A|401:O4(CIT)
    add a default BCOR for bond A|401:C6(CIT) A|401:O5(CIT)
    add a default BCOR for bond A|401:C6(CIT) A|401:O6(CIT)
    add a default BCOR for bond B|401:C01(LIG) B|401:C02(LIG)
    add a default BCOR for bond B|401:C02(LIG) B|401:C03(LIG)
    add a default BCOR for bond B|401:C02(LIG) B|401:C04(LIG)
    add a default BCOR for bond B|401:C04(LIG) B|401:N05(LIG)
    add a default BCOR for bond B|401:C04(LIG) B|401:C06(LIG)
    add a default BCOR for bond B|401:C06(LIG) B|401:O07(LIG)
    add a default BCOR for bond B|401:C06(LIG) B|401:N08(LIG)
    add a default BCOR for bond B|401:N08(LIG) B|401:C09(LIG)
    add a default BCOR for bond B|401:N08(LIG) B|401:C13(LIG)
    add a default BCOR for bond B|401:C09(LIG) B|401:C10(LIG)
    add a default BCOR for bond B|401:C10(LIG) B|401:O11(LIG)
    add a default BCOR for bond B|401:O11(LIG) B|401:C12(LIG)
    add a default BCOR for bond B|401:C12(LIG) B|401:C13(LIG)

 Have added a total of        25 BCORrels for bonds which do not already have a BCORrel


 No bonds (for potential BCORrel addition) were skipped due to both atoms having constant/fixed B(iso)


 ----------- Deactivate hydrogen-free planes if hydrogen atoms present

 A total of       0 planes have been deactivated because they are duplicated hydrogen-free and with-hydrogen forms.

 ----------- Handle peptide omega angle(s)

      Torsion angles split into peptide omega and other
         Number of peptide omega angles is       698
         Number of other torsion is             1270
      Peptide omega angles are monitored rather than directly restrained (PLANE used instead).

 ----------- Fix cis-PRO

 Now apply the fix to get correct restraints for cis PRO residues
   Have got      11 bonds records and      26 angles
   from GEOMETRY cards for the special PROC residue
   Weight for ideal-distance contact term=     4.000

 ----------- Prepare SSBOND/LINK for output PDB file


 ----------- Prepare for QM


 ----------- Prepare symmetry in utility distance restraints


 ############### Setting up Rhofit contacts (if needed)

 No ligand-(fixed protein) contact term will be used

 ############### Working out initial function and gradient


   MaxLik Scaling Cycle 0002
 
 Initial functional value = 4.368362E+05 rms gradient=      62.8969
 X-ray contribution to function value = 4.082081E+05
 Geometry contribution (inc ncs)      =      28628.1
 Geometry function summary
     term                           number     weight       rms        GooF      function     fn/numb
     ================================================================================================
     BOND bond lengths (angs)       5655       2.000      0.0048       0.238       639.51       0.113
     ANGL bond angles (degs)        7674       2.000        0.81       0.483      3584.50       0.467
     TORS split-harmonic tors       1270       0.000       16.53       1.040         0.00       0.000
     SINTOR sinusoidal tors         1270       2.000       16.53                  2099.14       1.653
     PLAN general plane (angs)       963       5.000      0.0111       0.553      1473.80       1.530
     BCORrelations (angs^2)         5655      10.000       0.757       0.270      4124.68       0.729
     CONT (bad contacts)               0       5.000                                 0.00
     IDEAL(ideal-dist contact)      5230       4.000                              7657.10
     CHIRAL (gelly semiharmon)       740       5.000                                27.16
     SIM similarity restraint for NCS and/or TARGET                               9022.22
     OCCSUM restraint on sum of occupanies                                           0.00       as none defined
     DISTAN utility restraints interatomic distance                                  0.00       as none defined
     UTILANGLE utility angle restraints                                              0.00       as none defined
     UTILTOR utility restraints on torsion angles                                    0.00       as none defined
     Peptide omega torsion angles                           2.33 degs - monitor only
 
 WARNING: Have |delta/sigma| deviations > 5.0 sigma. Number of outliers for each term: 
 WARNING:         1 bond angles.  Worst is     5.0 sigs   134.22 degs B|28:C.A(ASN)=B|29:N(ASN)=B|29:CA(ASN)

 Weighted rms      0.0048         0.811  (temporary rms bond and angle for BUSTER to pick up!)
 Weighted intra-residual rms(bond) for StdProtein (excluding H)      0.0046
 Weighted intra-residual rms(bond) for Buffer     (excluding H)      0.0036

 ############### Running initial check


 Initial Geometry Sanity Check

 Check for duplicate bonds: no duplicates found
 
 Check for duplicate or missing bond angles: all are OK
 Check for bond between an atom and itself not involving symmetry:  all are OK
   There are no bad contacts where Delta/sigma is greater than     6.5
   Worst contact dist=   2.843 std=   3.185 sigma=   0.107 abs(Delta/sigma)=    3.192 ats B|28:CB.B (ASN) B|29:N (ASN)
   It is likely that the geometry libraries, sequence info and links match the input molecule
 GEOMETRY CHECK OVERALL:  PASS


 ############### Creating geometry/screen report


 ############### Option "tao" - run Tao optimizer

 TAO: Toolkit for Advanced Optimization. Reference: 
     Munson, T., Sarich, J., Wild, S., Benson, S., McInnes, L.C. (2018)
     "TAO User Manual (Revision 3.9)", Mathematics and Computer Science
     Division, Argonne National Laboratory
     ANL/MCS-TM-322, http://www.mcs.anl.gov/petsc/
 
 Have found -keeppdb flag so will write out a .pdb file every 1000000 its during opt
 Will apply TAO optimization 
 Maximum number of iterations      100
 Convergence test on Grms       4.0000 (-glim)
 Limit on rms displacement to original position (rmsD2init): NONE test inactive (-dlim)

    analysing argument no 0 : -autoncs with j=0
    analysing argument no 1 : -hydrogens_input_occ2zero with j=0
    analysing argument no 2 : -hydrogens_output_occ2heavy with j=0
    analysing argument no 3 : -screen with j=0
    analysing argument no 4 : 100 with j=0
    analysing argument no 5 : -screen_sigma with j=0
    analysing argument no 6 : 3.0 with j=0
    analysing argument no 7 : -keeppdb with j=0
    analysing argument no 8 : 1000000 with j=0
    analysing argument no 9 : -taomonitor_verbose with j=0
    found argument starting with "-tao" : -taomonitor_verbose
    analysing argument no 10 : 2 with j=1
    found value after -tao/-options_/-viewer_ argument : "2"
 PETSC/TAO: setting option "-taomonitor_verbose" = "2"


   MaxLik Scaling Cycle 0003
   Iters     Tot_Funct    Xray_Funct    Geom_Funct          Grms     rmsD2init     maxD2init             R         Rfree           LLG       LLGfree     rms(bond)    rms(angle)       Opttag_H
   Iters             K   B_IMPF_FRAG        K_MISS   B_TEMP_MISS   K_IMPF_MISS   B_IMPF_MISS        K_SOLV   B_TEMP_SOLV   K_IMPF_SOLV   B_IMPF_SOLV        B_TEMP     B_TEMP_11     B_TEMP_12     B_TEMP_22     B_TEMP_13     B_TEMP_23     B_TEMP_33      K_WILSON      B_WILSON      FGCYCLES       Scatag_H
   Iters          BOND         ANGLE      IMPROPER       TORSION    SINTORSION      TRIGONAL        PLANAR  BCORRELATION       CONTACT     IDEALDIST        CHIRAL    SIMILARITY       OCCSSUM  UTILDISTANCE     UTILANGLE   UTILTORSION        RHOFIT        ADP-01        ADP-02        ADP-03        ADP-04        ADP-05        ADP-06        ADP-07        ADP-08        ADP-09        ADP-10      RHOFITPC            QM       Funtag_H
       0  4.368362E+05  4.082081E+05       28628.1       62.8969  0.000000E+00  0.000000E+00      0.199571      0.235406  0.000000E+00  0.000000E+00    0.00477153      0.810958       Opttag_D
       0       74.9550       1.90776       1.00000  0.000000E+00       1.00000  0.000000E+00      0.934926       66.2517       1.00000       34.5709  0.000000E+00       8.86001  0.000000E+00       8.86001  0.000000E+00  0.000000E+00      -17.7200       76.4175       3.81646        2       Scatag_D
       0       639.511       3584.50  0.000000E+00  0.000000E+00       2099.14  0.000000E+00       1473.80       4124.68  0.000000E+00       7657.10       27.1604       9022.22 -1.000000E+10 -1.000000E+10 -1.000000E+10 -1.000000E+10 -1.000000E+10  0.000000E+00  0.000000E+00  0.000000E+00  0.000000E+00  0.000000E+00  0.000000E+00  0.000000E+00  0.000000E+00  0.000000E+00  0.000000E+00  0.000000E+00  0.000000E+00       Funtag_D
   Geometry Restraint Deviations SCREEN Information

 

 An atom has moved more than     1.50 A from the initial/last
 position - if this was an accepted trial point, probably need to update the
 non-bonded contact list.


   MaxLik Scaling Cycle 0004

   MaxLik Scaling Cycle 0005

   MaxLik Scaling Cycle 0006

   Wilson plot

   Statistics in resolution bins after refinement cycle 0001
```


|  |  |
| --- | --- |
|  | - Rxpct factors plot- Rxpct factors table- <Log-Lik Gain>- Ln Sigma A- Correlation Coefficients |


```
   Iters     Tot_Funct    Xray_Funct    Geom_Funct          Grms     rmsD2init     maxD2init             R         Rfree           LLG       LLGfree     rms(bond)    rms(angle)
   Iters             K   B_IMPF_FRAG        K_MISS   B_TEMP_MISS   K_IMPF_MISS   B_IMPF_MISS        K_SOLV   B_TEMP_SOLV   K_IMPF_SOLV   B_IMPF_SOLV        B_TEMP     B_TEMP_11     B_TEMP_12     B_TEMP_22     B_TEMP_13     B_TEMP_23     B_TEMP_33      K_WILSON      B_WILSON      FGCYCLES
   Iters          BOND         ANGLE      IMPROPER       TORSION    SINTORSION      TRIGONAL        PLANAR  BCORRELATION       CONTACT     IDEALDIST        CHIRAL    SIMILARITY       OCCSSUM  UTILDISTANCE     UTILANGLE   UTILTORSION        RHOFIT        ADP-01        ADP-02        ADP-03        ADP-04        ADP-05        ADP-06        ADP-07        ADP-08        ADP-09        ADP-10      RHOFITPC            QM
       1  4.358990E+05  4.072986E+05       28600.4       57.0394  9.437609E-04    0.00604577      0.199037      0.235271    0.00256735 -4.004557E-04    0.00498025      0.813147       Opttag_D
       1       74.9138       1.86213       1.00000  0.000000E+00       1.00000  0.000000E+00      0.935041       65.9194       1.00000       34.5797  0.000000E+00       8.87151  0.000000E+00       8.87151  0.000000E+00  0.000000E+00      -17.7430       76.4175       3.81646        3       Scatag_D
       1       696.681       3603.88  0.000000E+00  0.000000E+00       2099.15  0.000000E+00       1484.32       3989.36  0.000000E+00       7666.69       27.7127       9032.61 -1.000000E+10 -1.000000E+10 -1.000000E+10 -1.000000E+10 -1.000000E+10  0.000000E+00  0.000000E+00  0.000000E+00  0.000000E+00  0.000000E+00  0.000000E+00  0.000000E+00  0.000000E+00  0.000000E+00  0.000000E+00  0.000000E+00  0.000000E+00       Funtag_D
   Geometry Restraint Deviations SCREEN Information

 

   MaxLik Scaling Cycle 0007

   Wilson plot

   Statistics in resolution bins after refinement cycle 0002
```


|  |  |
| --- | --- |
|  | - Rxpct factors plot- Rxpct factors table- <Log-Lik Gain>- Ln Sigma A- Correlation Coefficients |


```
   Iters     Tot_Funct    Xray_Funct    Geom_Funct          Grms     rmsD2init     maxD2init             R         Rfree           LLG       LLGfree     rms(bond)    rms(angle)
   Iters             K   B_IMPF_FRAG        K_MISS   B_TEMP_MISS   K_IMPF_MISS   B_IMPF_MISS        K_SOLV   B_TEMP_SOLV   K_IMPF_SOLV   B_IMPF_SOLV        B_TEMP     B_TEMP_11     B_TEMP_12     B_TEMP_22     B_TEMP_13     B_TEMP_23     B_TEMP_33      K_WILSON      B_WILSON      FGCYCLES
   Iters          BOND         ANGLE      IMPROPER       TORSION    SINTORSION      TRIGONAL        PLANAR  BCORRELATION       CONTACT     IDEALDIST        CHIRAL    SIMILARITY       OCCSSUM  UTILDISTANCE     UTILANGLE   UTILTORSION        RHOFIT        ADP-01        ADP-02        ADP-03        ADP-04        ADP-05        ADP-06        ADP-07        ADP-08        ADP-09        ADP-10      RHOFITPC            QM
       2  4.352096E+05  4.064783E+05       28731.3       44.5515    0.00196838     0.0131516      0.198514      0.235130    0.00488236 -9.033745E-04    0.00527769      0.816580       Opttag_D
       2       74.8774       1.82435       1.00000  0.000000E+00       1.00000  0.000000E+00      0.935056       65.7558       1.00000       34.5149  0.000000E+00       8.87741  0.000000E+00       8.87741  0.000000E+00  0.000000E+00      -17.7548       76.4175       3.81646        1       Scatag_D
       2       782.383       3634.37  0.000000E+00  0.000000E+00       2098.95  0.000000E+00       1499.39       3948.82  0.000000E+00       7684.16       28.1279       9055.10 -1.000000E+10 -1.000000E+10 -1.000000E+10 -1.000000E+10 -1.000000E+10  0.000000E+00  0.000000E+00  0.000000E+00  0.000000E+00  0.000000E+00  0.000000E+00  0.000000E+00  0.000000E+00  0.000000E+00  0.000000E+00  0.000000E+00  0.000000E+00       Funtag_D
   Geometry Restraint Deviations SCREEN Information

 
       3  4.345934E+05  4.051946E+05       29398.8       49.3244    0.00373233     0.0253161      0.197681      0.234894    0.00850505   -0.00174341    0.00581476      0.825554       Opttag_D
       3       74.8214       1.76802       1.00000  0.000000E+00       1.00000  0.000000E+00      0.935047       65.5697       1.00000       34.3772  0.000000E+00       8.88464  0.000000E+00       8.88464  0.000000E+00  0.000000E+00      -17.7693       76.4175       3.81646        1       Scatag_D
       3       949.722       3714.69  0.000000E+00  0.000000E+00       2098.84  0.000000E+00       1530.50       4241.41  0.000000E+00       7724.13       28.5895       9110.90 -1.000000E+10 -1.000000E+10 -1.000000E+10 -1.000000E+10 -1.000000E+10  0.000000E+00  0.000000E+00  0.000000E+00  0.000000E+00  0.000000E+00  0.000000E+00  0.000000E+00  0.000000E+00  0.000000E+00  0.000000E+00  0.000000E+00  0.000000E+00       Funtag_D
       4  4.341581E+05  4.038138E+05       30344.2       57.3198    0.00578252     0.0392385      0.196787      0.234632     0.0124020   -0.00264127    0.00641160      0.836713       Opttag_D
       4       74.7633       1.71022       1.00000  0.000000E+00       1.00000  0.000000E+00      0.935022       65.4242       1.00000       34.2034  0.000000E+00       8.89144  0.000000E+00       8.89144  0.000000E+00  0.000000E+00      -17.7829       76.4175       3.81646        1       Scatag_D
       4       1154.69       3815.79  0.000000E+00  0.000000E+00       2098.82  0.000000E+00       1574.03       4696.45  0.000000E+00       7781.78       28.7497       9193.92 -1.000000E+10 -1.000000E+10 -1.000000E+10 -1.000000E+10 -1.000000E+10  0.000000E+00  0.000000E+00  0.000000E+00  0.000000E+00  0.000000E+00  0.000000E+00  0.000000E+00  0.000000E+00  0.000000E+00  0.000000E+00  0.000000E+00  0.000000E+00       Funtag_D
       5  4.337533E+05  4.033039E+05       30449.4       38.6241    0.00666027     0.0449241      0.196452      0.234496     0.0138413   -0.00287168    0.00650782      0.833232       Opttag_D
       5       74.7421       1.69012       1.00000  0.000000E+00       1.00000  0.000000E+00      0.934987       65.4145       1.00000       34.1194  0.000000E+00       8.89406  0.000000E+00       8.89406  0.000000E+00  0.000000E+00      -17.7881       76.4175       3.81646        1       Scatag_D
       5       1189.60       3784.11  0.000000E+00  0.000000E+00       2098.85  0.000000E+00       1595.79       4718.71  0.000000E+00       7811.02       28.4417       9222.89 -1.000000E+10 -1.000000E+10 -1.000000E+10 -1.000000E+10 -1.000000E+10  0.000000E+00  0.000000E+00  0.000000E+00  0.000000E+00  0.000000E+00  0.000000E+00  0.000000E+00  0.000000E+00  0.000000E+00  0.000000E+00  0.000000E+00  0.000000E+00       Funtag_D
       6  4.331340E+05  4.020987E+05       31035.3       41.9558    0.00900121     0.0589445      0.195660      0.234141     0.0172425   -0.00329780    0.00675134      0.842530       Opttag_D
       6       74.6942       1.64433       1.00000  0.000000E+00       1.00000  0.000000E+00      0.934876       65.4416       1.00000       33.9044  0.000000E+00       8.89901  0.000000E+00       8.89901  0.000000E+00  0.000000E+00      -17.7980       76.4175       3.81646        1       Scatag_D
       6       1280.30       3869.04  0.000000E+00  0.000000E+00       2099.05  0.000000E+00       1656.86       4914.40  0.000000E+00       7892.79       27.9205       9294.98 -1.000000E+10 -1.000000E+10 -1.000000E+10 -1.000000E+10 -1.000000E+10  0.000000E+00  0.000000E+00  0.000000E+00  0.000000E+00  0.000000E+00  0.000000E+00  0.000000E+00  0.000000E+00  0.000000E+00  0.000000E+00  0.000000E+00  0.000000E+00       Funtag_D
       7  4.327163E+05  4.009490E+05       31767.3       58.4986     0.0114911     0.0725502      0.194891      0.233782     0.0204871   -0.00367566    0.00701030      0.859868       Opttag_D
       7       74.6526       1.60233       1.00000  0.000000E+00       1.00000  0.000000E+00      0.934741       65.5053       1.00000       33.6934  0.000000E+00       8.90110  0.000000E+00       8.90110  0.000000E+00  0.000000E+00      -17.8022       76.4175       3.81646        1       Scatag_D
       7       1380.40       4029.92  0.000000E+00  0.000000E+00       2099.44  0.000000E+00       1724.81       5153.83  0.000000E+00       7985.00       28.1485       9365.77 -1.000000E+10 -1.000000E+10 -1.000000E+10 -1.000000E+10 -1.000000E+10  0.000000E+00  0.000000E+00  0.000000E+00  0.000000E+00  0.000000E+00  0.000000E+00  0.000000E+00  0.000000E+00  0.000000E+00  0.000000E+00  0.000000E+00  0.000000E+00       Funtag_D
       8  4.321994E+05  3.996982E+05       32501.3       42.0121     0.0144322     0.0876025      0.194057      0.233375     0.0240173   -0.00409338    0.00724229      0.863774       Opttag_D
       8       74.6111       1.55804       1.00000  0.000000E+00       1.00000  0.000000E+00      0.934578       65.5945       1.00000       33.4669  0.000000E+00       8.90173  0.000000E+00       8.90173  0.000000E+00  0.000000E+00      -17.8035       76.4175       3.81646        1       Scatag_D
       8       1473.28       4066.60  0.000000E+00  0.000000E+00       2099.98  0.000000E+00       1808.45       5481.33  0.000000E+00       8098.45       28.9168       9444.25 -1.000000E+10 -1.000000E+10 -1.000000E+10 -1.000000E+10 -1.000000E+10  0.000000E+00  0.000000E+00  0.000000E+00  0.000000E+00  0.000000E+00  0.000000E+00  0.000000E+00  0.000000E+00  0.000000E+00  0.000000E+00  0.000000E+00  0.000000E+00       Funtag_D
       9  4.316922E+05  3.984378E+05       33254.4       45.9276     0.0177810      0.102596      0.193216      0.232917     0.0275744   -0.00444414    0.00739918      0.878128       Opttag_D
       9       74.5738       1.51456       1.00000  0.000000E+00       1.00000  0.000000E+00      0.934383       65.7023       1.00000       33.2543  0.000000E+00       8.90184  0.000000E+00       8.90184  0.000000E+00  0.000000E+00      -17.8037       76.4175       3.81646        1       Scatag_D
       9       1537.80       4202.88  0.000000E+00  0.000000E+00       2100.84  0.000000E+00       1899.67       5749.72  0.000000E+00       8231.98       30.5696       9500.95 -1.000000E+10 -1.000000E+10 -1.000000E+10 -1.000000E+10 -1.000000E+10  0.000000E+00  0.000000E+00  0.000000E+00  0.000000E+00  0.000000E+00  0.000000E+00  0.000000E+00  0.000000E+00  0.000000E+00  0.000000E+00  0.000000E+00  0.000000E+00       Funtag_D
      10  4.312209E+05  3.974730E+05       33747.9       41.3910     0.0207411      0.114055      0.192574      0.232519     0.0302971   -0.00464111    0.00742764      0.884771       Opttag_D
      10       74.5488       1.48206       1.00000  0.000000E+00       1.00000  0.000000E+00      0.934199       65.8043       1.00000       33.1026  0.000000E+00       8.90201  0.000000E+00       8.90201  0.000000E+00  0.000000E+00      -17.8040       76.4175       3.81646        1       Scatag_D
      10       1549.65       4266.71  0.000000E+00  0.000000E+00       2101.94  0.000000E+00       1971.68       5952.29  0.000000E+00       8349.42       32.5388       9523.66 -1.000000E+10 -1.000000E+10 -1.000000E+10 -1.000000E+10 -1.000000E+10  0.000000E+00  0.000000E+00  0.000000E+00  0.000000E+00  0.000000E+00  0.000000E+00  0.000000E+00  0.000000E+00  0.000000E+00  0.000000E+00  0.000000E+00  0.000000E+00       Funtag_D
      11  4.307812E+05  3.968329E+05       33948.3       37.3193     0.0230599      0.121616      0.192145      0.232212     0.0321036   -0.00472153    0.00737875      0.889496       Opttag_D
      11       74.5333       1.46059       1.00000  0.000000E+00       1.00000  0.000000E+00      0.934038       65.8861       1.00000       33.0097  0.000000E+00       8.90453  0.000000E+00       8.90453  0.000000E+00  0.000000E+00      -17.8091       76.4175       3.81646        1       Scatag_D
      11       1529.32       4312.41  0.000000E+00  0.000000E+00       2102.86  0.000000E+00       2013.54       6000.44  0.000000E+00       8438.86       33.8833       9516.99 -1.000000E+10 -1.000000E+10 -1.000000E+10 -1.000000E+10 -1.000000E+10  0.000000E+00  0.000000E+00  0.000000E+00  0.000000E+00  0.000000E+00  0.000000E+00  0.000000E+00  0.000000E+00  0.000000E+00  0.000000E+00  0.000000E+00  0.000000E+00       Funtag_D
      12  4.303042E+05  3.957377E+05       34566.4       58.2481     0.0277254      0.143402      0.191415      0.231658     0.0351944   -0.00475776    0.00733823      0.908911       Opttag_D
      12       74.5098       1.42447       1.00000  0.000000E+00       1.00000  0.000000E+00      0.933710       66.0611       1.00000       32.8501  0.000000E+00       8.91256  0.000000E+00       8.91256  0.000000E+00  0.000000E+00      -17.8251       76.4175       3.81646        1       Scatag_D
      12       1512.57       4502.72  0.000000E+00  0.000000E+00       2105.51  0.000000E+00       2081.11       6200.03  0.000000E+00       8620.15       35.8164       9508.53 -1.000000E+10 -1.000000E+10 -1.000000E+10 -1.000000E+10 -1.000000E+10  0.000000E+00  0.000000E+00  0.000000E+00  0.000000E+00  0.000000E+00  0.000000E+00  0.000000E+00  0.000000E+00  0.000000E+00  0.000000E+00  0.000000E+00  0.000000E+00       Funtag_D
      13  4.298922E+05  3.950978E+05       34794.3       40.6924     0.0302977      0.157580      0.190984      0.231359     0.0370003   -0.00487314    0.00733047      0.910845       Opttag_D
      13       74.4958       1.40399       1.00000  0.000000E+00       1.00000  0.000000E+00      0.933535       66.1714       1.00000       32.7365  0.000000E+00       8.91611  0.000000E+00       8.91611  0.000000E+00  0.000000E+00      -17.8322       76.4175       3.81646        1       Scatag_D
      13       1509.37       4521.90  0.000000E+00  0.000000E+00       2107.15  0.000000E+00       2120.67       6244.03  0.000000E+00       8725.66       36.1284       9529.41 -1.000000E+10 -1.000000E+10 -1.000000E+10 -1.000000E+10 -1.000000E+10  0.000000E+00  0.000000E+00  0.000000E+00  0.000000E+00  0.000000E+00  0.000000E+00  0.000000E+00  0.000000E+00  0.000000E+00  0.000000E+00  0.000000E+00  0.000000E+00       Funtag_D
      14  4.296130E+05  3.948293E+05       34783.7       32.8933     0.0314180      0.163861      0.190802      0.231230     0.0377583   -0.00487775    0.00731910      0.911067       Opttag_D
      14       74.4891       1.39529       1.00000  0.000000E+00       1.00000  0.000000E+00      0.933445       66.2232       1.00000       32.6845  0.000000E+00       8.91923  0.000000E+00       8.91923  0.000000E+00  0.000000E+00      -17.8385       76.4175       3.81646        1       Scatag_D
      14       1504.69       4524.10  0.000000E+00  0.000000E+00       2107.95  0.000000E+00       2126.39       6182.46  0.000000E+00       8769.31       35.8451       9533.00 -1.000000E+10 -1.000000E+10 -1.000000E+10 -1.000000E+10 -1.000000E+10  0.000000E+00  0.000000E+00  0.000000E+00  0.000000E+00  0.000000E+00  0.000000E+00  0.000000E+00  0.000000E+00  0.000000E+00  0.000000E+00  0.000000E+00  0.000000E+00       Funtag_D
      15  4.292089E+05  3.939618E+05       35247.1       36.3775     0.0351340      0.184139      0.190226      0.230831     0.0402066   -0.00496287    0.00737499      0.919534       Opttag_D
      15       74.4725       1.36776       1.00000  0.000000E+00       1.00000  0.000000E+00      0.933174       66.3818       1.00000       32.5346  0.000000E+00       8.92467  0.000000E+00       8.92467  0.000000E+00  0.000000E+00      -17.8493       76.4175       3.81646        1       Scatag_D
      15       1527.76       4608.58  0.000000E+00  0.000000E+00       2111.12  0.000000E+00       2160.17       6318.57  0.000000E+00       8921.56       36.4085       9562.90 -1.000000E+10 -1.000000E+10 -1.000000E+10 -1.000000E+10 -1.000000E+10  0.000000E+00  0.000000E+00  0.000000E+00  0.000000E+00  0.000000E+00  0.000000E+00  0.000000E+00  0.000000E+00  0.000000E+00  0.000000E+00  0.000000E+00  0.000000E+00       Funtag_D
      16  4.290132E+05  3.927191E+05       36294.1       69.8302     0.0409466      0.215107      0.189407      0.230304     0.0437135   -0.00515641    0.00753595      0.948889       Opttag_D
      16       74.4567       1.32908       1.00000  0.000000E+00       1.00000  0.000000E+00      0.932775       66.5987       1.00000       32.3559  0.000000E+00       8.92942  0.000000E+00       8.92942  0.000000E+00  0.000000E+00      -17.8588       76.4175       3.81646        1       Scatag_D
      16       1595.17       4907.53  0.000000E+00  0.000000E+00       2117.01  0.000000E+00       2224.48       6621.74  0.000000E+00       9169.33       38.4915       9620.31 -1.000000E+10 -1.000000E+10 -1.000000E+10 -1.000000E+10 -1.000000E+10  0.000000E+00  0.000000E+00  0.000000E+00  0.000000E+00  0.000000E+00  0.000000E+00  0.000000E+00  0.000000E+00  0.000000E+00  0.000000E+00  0.000000E+00  0.000000E+00       Funtag_D
      17  4.286784E+05  3.925713E+05       36107.1       32.3308     0.0415956      0.218520      0.189315      0.230262     0.0441308   -0.00520990    0.00749491      0.934798       Opttag_D
      17       74.4560       1.32431       1.00000  0.000000E+00       1.00000  0.000000E+00      0.932731       66.6112       1.00000       32.3457  0.000000E+00       8.92998  0.000000E+00       8.92998  0.000000E+00  0.000000E+00      -17.8600       76.4175       3.81646        1       Scatag_D
      17       1577.85       4762.86  0.000000E+00  0.000000E+00       2117.77  0.000000E+00       2228.54       6566.78  0.000000E+00       9195.06       39.0278       9619.19 -1.000000E+10 -1.000000E+10 -1.000000E+10 -1.000000E+10 -1.000000E+10  0.000000E+00  0.000000E+00  0.000000E+00  0.000000E+00  0.000000E+00  0.000000E+00  0.000000E+00  0.000000E+00  0.000000E+00  0.000000E+00  0.000000E+00  0.000000E+00       Funtag_D
      18  4.284392E+05  3.921320E+05       36307.3       24.1658     0.0437174      0.229584      0.189032      0.230108     0.0453706   -0.00533558    0.00752223      0.938464       Opttag_D
      18       74.4538       1.31061       1.00000  0.000000E+00       1.00000  0.000000E+00      0.932587       66.6730       1.00000       32.3020  0.000000E+00       8.93165  0.000000E+00       8.93165  0.000000E+00  0.000000E+00      -17.8633       76.4175       3.81646        1       Scatag_D
      18       1589.37       4800.29  0.000000E+00  0.000000E+00       2120.53  0.000000E+00       2248.71       6591.57  0.000000E+00       9284.36       40.3595       9632.08 -1.000000E+10 -1.000000E+10 -1.000000E+10 -1.000000E+10 -1.000000E+10  0.000000E+00  0.000000E+00  0.000000E+00  0.000000E+00  0.000000E+00  0.000000E+00  0.000000E+00  0.000000E+00  0.000000E+00  0.000000E+00  0.000000E+00  0.000000E+00       Funtag_D
      19  4.282220E+05  3.914852E+05       36736.8       29.7317     0.0471050      0.247018      0.188614      0.229888     0.0471960   -0.00551178    0.00757812      0.947441       Opttag_D
      19       74.4519       1.29081       1.00000  0.000000E+00       1.00000  0.000000E+00      0.932349       66.7874       1.00000       32.2287  0.000000E+00       8.93568  0.000000E+00       8.93568  0.000000E+00  0.000000E+00      -17.8714       76.4175       3.81646        1       Scatag_D
      19       1613.08       4892.57  0.000000E+00  0.000000E+00       2125.14  0.000000E+00       2280.43       6696.93  0.000000E+00       9428.51       41.9959       9658.19 -1.000000E+10 -1.000000E+10 -1.000000E+10 -1.000000E+10 -1.000000E+10  0.000000E+00  0.000000E+00  0.000000E+00  0.000000E+00  0.000000E+00  0.000000E+00  0.000000E+00  0.000000E+00  0.000000E+00  0.000000E+00  0.000000E+00  0.000000E+00       Funtag_D
      20  4.279959E+05  3.904359E+05       37560.0       52.3841     0.0531364      0.277266      0.187943      0.229575     0.0501572   -0.00580263    0.00769379      0.966427       Opttag_D
      20       74.4550       1.25906       1.00000  0.000000E+00       1.00000  0.000000E+00      0.931915       66.9911       1.00000       32.1198  0.000000E+00       8.94551  0.000000E+00       8.94551  0.000000E+00  0.000000E+00      -17.8910       76.4175       3.81646        1       Scatag_D
      20       1662.69       5090.61  0.000000E+00  0.000000E+00       2135.01  0.000000E+00       2332.70       6905.84  0.000000E+00       9684.39       44.8059       9703.94 -1.000000E+10 -1.000000E+10 -1.000000E+10 -1.000000E+10 -1.000000E+10  0.000000E+00  0.000000E+00  0.000000E+00  0.000000E+00  0.000000E+00  0.000000E+00  0.000000E+00  0.000000E+00  0.000000E+00  0.000000E+00  0.000000E+00  0.000000E+00       Funtag_D
      21  4.277053E+05  3.901031E+05       37602.2       29.4263     0.0552538      0.287412      0.187732      0.229465     0.0510964   -0.00583109    0.00768275      0.961596       Opttag_D
      21       74.4592       1.24888       1.00000  0.000000E+00       1.00000  0.000000E+00      0.931753       67.0601       1.00000       32.0892  0.000000E+00       8.95186  0.000000E+00       8.95186  0.000000E+00  0.000000E+00      -17.9037       76.4175       3.81646        1       Scatag_D
      21       1657.93       5039.85  0.000000E+00  0.000000E+00       2138.79  0.000000E+00       2345.47       6889.25  0.000000E+00       9769.27       45.4584       9716.21 -1.000000E+10 -1.000000E+10 -1.000000E+10 -1.000000E+10 -1.000000E+10  0.000000E+00  0.000000E+00  0.000000E+00  0.000000E+00  0.000000E+00  0.000000E+00  0.000000E+00  0.000000E+00  0.000000E+00  0.000000E+00  0.000000E+00  0.000000E+00       Funtag_D
      22  4.274545E+05  3.898993E+05       37555.2       28.5553     0.0566706      0.293586      0.187600      0.229385     0.0516717   -0.00576035    0.00766555      0.961930       Opttag_D
      22       74.4654       1.24227       1.00000  0.000000E+00       1.00000  0.000000E+00      0.931633       67.0984       1.00000       32.0783  0.000000E+00       8.95986  0.000000E+00       8.95986  0.000000E+00  0.000000E+00      -17.9197       76.4175       3.81646        1       Scatag_D
      22       1650.51       5043.35  0.000000E+00  0.000000E+00       2142.09  0.000000E+00       2345.09       6794.94  0.000000E+00       9815.27       45.5765       9718.42 -1.000000E+10 -1.000000E+10 -1.000000E+10 -1.000000E+10 -1.000000E+10  0.000000E+00  0.000000E+00  0.000000E+00  0.000000E+00  0.000000E+00  0.000000E+00  0.000000E+00  0.000000E+00  0.000000E+00  0.000000E+00  0.000000E+00  0.000000E+00       Funtag_D
      23  4.271920E+05  3.895227E+05       37669.3       34.8967     0.0593359      0.307573      0.187362      0.229232     0.0527346   -0.00567559    0.00768683      0.964619       Opttag_D
      23       74.4793       1.23024       1.00000  0.000000E+00       1.00000  0.000000E+00      0.931421       67.1653       1.00000       32.0669  0.000000E+00       8.97218  0.000000E+00       8.97218  0.000000E+00  0.000000E+00      -17.9444       76.4175       3.81646        1       Scatag_D
      23       1659.69       5071.58  0.000000E+00  0.000000E+00       2148.07  0.000000E+00       2351.63       6752.87  0.000000E+00       9906.43       46.2768       9732.76 -1.000000E+10 -1.000000E+10 -1.000000E+10 -1.000000E+10 -1.000000E+10  0.000000E+00  0.000000E+00  0.000000E+00  0.000000E+00  0.000000E+00  0.000000E+00  0.000000E+00  0.000000E+00  0.000000E+00  0.000000E+00  0.000000E+00  0.000000E+00       Funtag_D
      24  4.269547E+05  3.889530E+05       38001.7       47.3756     0.0636351      0.333993      0.187012      0.229005     0.0543424   -0.00563629    0.00775031      0.972323       Opttag_D
      24       74.5081       1.21234       1.00000  0.000000E+00       1.00000  0.000000E+00      0.931094       67.2634       1.00000       32.0706  0.000000E+00       8.98796  0.000000E+00       8.98796  0.000000E+00  0.000000E+00      -17.9759       76.4175       3.81646        1       Scatag_D
      24       1687.21       5152.91  0.000000E+00  0.000000E+00       2157.79  0.000000E+00       2369.05       6768.75  0.000000E+00       10054.5       47.6872       9763.78 -1.000000E+10 -1.000000E+10 -1.000000E+10 -1.000000E+10 -1.000000E+10  0.000000E+00  0.000000E+00  0.000000E+00  0.000000E+00  0.000000E+00  0.000000E+00  0.000000E+00  0.000000E+00  0.000000E+00  0.000000E+00  0.000000E+00  0.000000E+00       Funtag_D
      25  4.267199E+05  3.887771E+05       37942.9       26.2833     0.0649415      0.342214      0.186910      0.228939     0.0548389   -0.00562834    0.00774560      0.966463       Opttag_D
      25       74.5210       1.20678       1.00000  0.000000E+00       1.00000  0.000000E+00      0.930996       67.2838       1.00000       32.0863  0.000000E+00       8.99187  0.000000E+00       8.99187  0.000000E+00  0.000000E+00      -17.9837       76.4175       3.81646        1       Scatag_D
      25       1685.17       5090.99  0.000000E+00  0.000000E+00       2160.51  0.000000E+00       2374.22       6721.27  0.000000E+00       10095.5       48.0790       9767.12 -1.000000E+10 -1.000000E+10 -1.000000E+10 -1.000000E+10 -1.000000E+10  0.000000E+00  0.000000E+00  0.000000E+00  0.000000E+00  0.000000E+00  0.000000E+00  0.000000E+00  0.000000E+00  0.000000E+00  0.000000E+00  0.000000E+00  0.000000E+00       Funtag_D
      26  4.265275E+05  3.885575E+05       37970.0       23.5110     0.0666138      0.352275      0.186785      0.228861     0.0554585   -0.00561026    0.00775391      0.967326       Opttag_D
      26       74.5397       1.19999       1.00000  0.000000E+00       1.00000  0.000000E+00      0.930873       67.3104       1.00000       32.1121  0.000000E+00       8.99561  0.000000E+00       8.99561  0.000000E+00  0.000000E+00      -17.9912       76.4175       3.81646        1       Scatag_D
      26       1688.78       5100.09  0.000000E+00  0.000000E+00       2163.79  0.000000E+00       2381.44       6669.35  0.000000E+00       10148.1       48.6221       9769.88 -1.000000E+10 -1.000000E+10 -1.000000E+10 -1.000000E+10 -1.000000E+10  0.000000E+00  0.000000E+00  0.000000E+00  0.000000E+00  0.000000E+00  0.000000E+00  0.000000E+00  0.000000E+00  0.000000E+00  0.000000E+00  0.000000E+00  0.000000E+00       Funtag_D
      27  4.263488E+05  3.882995E+05       38049.3       24.9850     0.0691149      0.366749      0.186638      0.228768     0.0561866   -0.00549732    0.00776173      0.969515       Opttag_D
      27       74.5706       1.19235       1.00000  0.000000E+00       1.00000  0.000000E+00      0.930680       67.3694       1.00000       32.1514  0.000000E+00       9.00127  0.000000E+00       9.00127  0.000000E+00  0.000000E+00      -18.0025       76.4175       3.81646        1       Scatag_D
      27       1692.19       5123.20  0.000000E+00  0.000000E+00       2168.83  0.000000E+00       2391.72       6623.90  0.000000E+00       10226.0       49.1806       9774.27 -1.000000E+10 -1.000000E+10 -1.000000E+10 -1.000000E+10 -1.000000E+10  0.000000E+00  0.000000E+00  0.000000E+00  0.000000E+00  0.000000E+00  0.000000E+00  0.000000E+00  0.000000E+00  0.000000E+00  0.000000E+00  0.000000E+00  0.000000E+00       Funtag_D
      28  4.261963E+05  3.879483E+05       38248.0       46.4882     0.0738980      0.394493      0.186441      0.228629     0.0571779   -0.00509632    0.00776421      0.978852       Opttag_D
      28       74.6409       1.18279       1.00000  0.000000E+00       1.00000  0.000000E+00      0.930309       67.5130       1.00000       32.2424  0.000000E+00       9.01012  0.000000E+00       9.01012  0.000000E+00  0.000000E+00      -18.0202       76.4175       3.81646        1       Scatag_D
      28       1693.27       5222.35  0.000000E+00  0.000000E+00       2178.82  0.000000E+00       2411.25       6540.93  0.000000E+00       10373.1       49.8777       9778.45 -1.000000E+10 -1.000000E+10 -1.000000E+10 -1.000000E+10 -1.000000E+10  0.000000E+00  0.000000E+00  0.000000E+00  0.000000E+00  0.000000E+00  0.000000E+00  0.000000E+00  0.000000E+00  0.000000E+00  0.000000E+00  0.000000E+00  0.000000E+00       Funtag_D
      29  4.259854E+05  3.879197E+05       38065.7       20.3133     0.0748708      0.399589      0.186423      0.228594     0.0572584   -0.00488363    0.00769554      0.971752       Opttag_D
      29       74.6598       1.18252       1.00000  0.000000E+00       1.00000  0.000000E+00      0.930227       67.5597       1.00000       32.2648  0.000000E+00       9.01139  0.000000E+00       9.01139  0.000000E+00  0.000000E+00      -18.0228       76.4175       3.81646        1       Scatag_D
      29       1663.45       5146.86  0.000000E+00  0.000000E+00       2180.81  0.000000E+00       2412.64       6442.89  0.000000E+00       10398.0       49.9785       9771.06 -1.000000E+10 -1.000000E+10 -1.000000E+10 -1.000000E+10 -1.000000E+10  0.000000E+00  0.000000E+00  0.000000E+00  0.000000E+00  0.000000E+00  0.000000E+00  0.000000E+00  0.000000E+00  0.000000E+00  0.000000E+00  0.000000E+00  0.000000E+00       Funtag_D
      30  4.258519E+05  3.879163E+05       37935.6       18.7722     0.0757678      0.404326      0.186419      0.228564     0.0572680   -0.00461102    0.00763857      0.971672       Opttag_D
      30       74.6819       1.18325       1.00000  0.000000E+00       1.00000  0.000000E+00      0.930149       67.6147       1.00000       32.2889  0.000000E+00       9.01164  0.000000E+00       9.01164  0.000000E+00  0.000000E+00      -18.0233       76.4175       3.81646        1       Scatag_D
      30       1638.91       5146.02  0.000000E+00  0.000000E+00       2182.77  0.000000E+00       2411.83       6329.00  0.000000E+00       10416.7       50.1497       9760.14 -1.000000E+10 -1.000000E+10 -1.000000E+10 -1.000000E+10 -1.000000E+10  0.000000E+00  0.000000E+00  0.000000E+00  0.000000E+00  0.000000E+00  0.000000E+00  0.000000E+00  0.000000E+00  0.000000E+00  0.000000E+00  0.000000E+00  0.000000E+00       Funtag_D
      31  4.257095E+05  3.878456E+05       37863.9       21.1837     0.0777179      0.420639      0.186376      0.228501     0.0574677   -0.00421653    0.00758108      0.972414       Opttag_D
      31       74.7253       1.18257       1.00000  0.000000E+00       1.00000  0.000000E+00      0.929999       67.7099       1.00000       32.3375  0.000000E+00       9.01157  0.000000E+00       9.01157  0.000000E+00  0.000000E+00      -18.0231       76.4175       3.81646        1       Scatag_D
      31       1614.34       5153.88  0.000000E+00  0.000000E+00       2186.91  0.000000E+00       2415.26       6227.88  0.000000E+00       10464.2       50.8975       9750.51 -1.000000E+10 -1.000000E+10 -1.000000E+10 -1.000000E+10 -1.000000E+10  0.000000E+00  0.000000E+00  0.000000E+00  0.000000E+00  0.000000E+00  0.000000E+00  0.000000E+00  0.000000E+00  0.000000E+00  0.000000E+00  0.000000E+00  0.000000E+00       Funtag_D
      32  4.256596E+05  3.876132E+05       38046.3       55.8033     0.0817786      0.459260      0.186242      0.228370     0.0581234   -0.00367060    0.00759422      0.986627       Opttag_D
      32       74.8188       1.17874       1.00000  0.000000E+00       1.00000  0.000000E+00      0.929731       67.8885       1.00000       32.4372  0.000000E+00       9.00756  0.000000E+00       9.00756  0.000000E+00  0.000000E+00      -18.0151       76.4175       3.81646        1       Scatag_D
      32       1619.94       5305.64  0.000000E+00  0.000000E+00       2196.79  0.000000E+00       2427.68       6136.07  0.000000E+00       10561.3       52.5522       9746.35 -1.000000E+10 -1.000000E+10 -1.000000E+10 -1.000000E+10 -1.000000E+10  0.000000E+00  0.000000E+00  0.000000E+00  0.000000E+00  0.000000E+00  0.000000E+00  0.000000E+00  0.000000E+00  0.000000E+00  0.000000E+00  0.000000E+00  0.000000E+00       Funtag_D
      33  4.254421E+05  3.875479E+05       37894.2       19.8570     0.0824487      0.465561      0.186204      0.228347     0.0583078   -0.00360871    0.00753472      0.974187       Opttag_D
      33       74.8332       1.17721       1.00000  0.000000E+00       1.00000  0.000000E+00      0.929695       67.9101       1.00000       32.4502  0.000000E+00       9.00690  0.000000E+00       9.00690  0.000000E+00  0.000000E+00      -18.0138       76.4175       3.81646        1       Scatag_D
      33       1594.65       5172.70  0.000000E+00  0.000000E+00       2197.67  0.000000E+00       2430.84       6121.80  0.000000E+00       10577.8       52.8059       9746.00 -1.000000E+10 -1.000000E+10 -1.000000E+10 -1.000000E+10 -1.000000E+10  0.000000E+00  0.000000E+00  0.000000E+00  0.000000E+00  0.000000E+00  0.000000E+00  0.000000E+00  0.000000E+00  0.000000E+00  0.000000E+00  0.000000E+00  0.000000E+00       Funtag_D
      34  4.253510E+05  3.874281E+05       37922.9       13.8459     0.0832025      0.473540      0.186134      0.228311     0.0586460   -0.00361142    0.00755212      0.974118       Opttag_D
      34       74.8507       1.17417       1.00000  0.000000E+00       1.00000  0.000000E+00      0.929666       67.9287       1.00000       32.4618  0.000000E+00       9.00532  0.000000E+00       9.00532  0.000000E+00  0.000000E+00      -18.0106       76.4175       3.81646        1       Scatag_D
      34       1602.03       5171.96  0.000000E+00  0.000000E+00       2199.17  0.000000E+00       2434.51       6119.56  0.000000E+00       10593.7       52.9240       9749.10 -1.000000E+10 -1.000000E+10 -1.000000E+10 -1.000000E+10 -1.000000E+10  0.000000E+00  0.000000E+00  0.000000E+00  0.000000E+00  0.000000E+00  0.000000E+00  0.000000E+00  0.000000E+00  0.000000E+00  0.000000E+00  0.000000E+00  0.000000E+00       Funtag_D
      35  4.252281E+05  3.871530E+05       38075.1       18.8354     0.0853270      0.495706      0.185971      0.228224     0.0594223   -0.00354709    0.00760363      0.976628       Opttag_D
      35       74.9011       1.16767       1.00000  0.000000E+00       1.00000  0.000000E+00      0.929575       67.9944       1.00000       32.4969  0.000000E+00       9.00109  0.000000E+00       9.00109  0.000000E+00  0.000000E+00      -18.0022       76.4175       3.81646        1       Scatag_D
      35       1623.95       5198.65  0.000000E+00  0.000000E+00       2203.88  0.000000E+00       2445.27       6153.24  0.000000E+00       10638.9       53.1680       9758.04 -1.000000E+10 -1.000000E+10 -1.000000E+10 -1.000000E+10 -1.000000E+10  0.000000E+00  0.000000E+00  0.000000E+00  0.000000E+00  0.000000E+00  0.000000E+00  0.000000E+00  0.000000E+00  0.000000E+00  0.000000E+00  0.000000E+00  0.000000E+00       Funtag_D
      36  4.251154E+05  3.867438E+05       38371.7       34.2019     0.0884407      0.529289      0.185724      0.228089     0.0605773   -0.00340938    0.00770544      0.982490       Opttag_D
      36       74.9769       1.15840       1.00000  0.000000E+00       1.00000  0.000000E+00      0.929453       68.1016       1.00000       32.5333  0.000000E+00       8.99508  0.000000E+00       8.99508  0.000000E+00  0.000000E+00      -17.9902       76.4175       3.81646        1       Scatag_D
      36       1667.73       5261.24  0.000000E+00  0.000000E+00       2210.96  0.000000E+00       2463.09       6237.56  0.000000E+00       10703.1       52.5936       9775.41 -1.000000E+10 -1.000000E+10 -1.000000E+10 -1.000000E+10 -1.000000E+10  0.000000E+00  0.000000E+00  0.000000E+00  0.000000E+00  0.000000E+00  0.000000E+00  0.000000E+00  0.000000E+00  0.000000E+00  0.000000E+00  0.000000E+00  0.000000E+00       Funtag_D
      37  4.249830E+05  3.864644E+05       38518.6       18.9509     0.0909145      0.555460      0.185554      0.227992     0.0613658   -0.00326843    0.00774683      0.981443       Opttag_D
      37       75.0375       1.15246       1.00000  0.000000E+00       1.00000  0.000000E+00      0.929354       68.1923       1.00000       32.5661  0.000000E+00       8.98995  0.000000E+00       8.98995  0.000000E+00  0.000000E+00      -17.9799       76.4175       3.81646        1       Scatag_D
      37       1685.70       5250.03  0.000000E+00  0.000000E+00       2216.95  0.000000E+00       2478.67       6291.00  0.000000E+00       10754.8       52.5546       9788.93 -1.000000E+10 -1.000000E+10 -1.000000E+10 -1.000000E+10 -1.000000E+10  0.000000E+00  0.000000E+00  0.000000E+00  0.000000E+00  0.000000E+00  0.000000E+00  0.000000E+00  0.000000E+00  0.000000E+00  0.000000E+00  0.000000E+00  0.000000E+00       Funtag_D
      38  4.248886E+05  3.862808E+05       38607.8       12.9290     0.0924194      0.572586      0.185438      0.227905     0.0618839   -0.00313696    0.00777041      0.982089       Opttag_D
      38       75.0761       1.14877       1.00000  0.000000E+00       1.00000  0.000000E+00      0.929299       68.2559       1.00000       32.5762  0.000000E+00       8.98672  0.000000E+00       8.98672  0.000000E+00  0.000000E+00      -17.9734       76.4175       3.81646        1       Scatag_D
      38       1695.98       5256.95  0.000000E+00  0.000000E+00       2220.61  0.000000E+00       2487.93       6316.63  0.000000E+00       10781.4       52.4737       9795.79 -1.000000E+10 -1.000000E+10 -1.000000E+10 -1.000000E+10 -1.000000E+10  0.000000E+00  0.000000E+00  0.000000E+00  0.000000E+00  0.000000E+00  0.000000E+00  0.000000E+00  0.000000E+00  0.000000E+00  0.000000E+00  0.000000E+00  0.000000E+00       Funtag_D
      39  4.247962E+05  3.860577E+05       38738.4       17.8712     0.0942291      0.594111      0.185296      0.227794     0.0625134   -0.00294812    0.00779718      0.984244       Opttag_D
      39       75.1242       1.14455       1.00000  0.000000E+00       1.00000  0.000000E+00      0.929236       68.3406       1.00000       32.5827  0.000000E+00       8.98248  0.000000E+00       8.98248  0.000000E+00  0.000000E+00      -17.9650       76.4175       3.81646        1       Scatag_D
      39       1707.69       5280.04  0.000000E+00  0.000000E+00       2225.06  0.000000E+00       2498.11       6362.13  0.000000E+00       10811.3       52.4918       9801.61 -1.000000E+10 -1.000000E+10 -1.000000E+10 -1.000000E+10 -1.000000E+10  0.000000E+00  0.000000E+00  0.000000E+00  0.000000E+00  0.000000E+00  0.000000E+00  0.000000E+00  0.000000E+00  0.000000E+00  0.000000E+00  0.000000E+00  0.000000E+00       Funtag_D
      40  4.247033E+05  3.857857E+05       38917.6       21.2588     0.0966120      0.622540      0.185127      0.227657     0.0632811   -0.00275142    0.00782404      0.986549       Opttag_D
      40       75.1884       1.13975       1.00000  0.000000E+00       1.00000  0.000000E+00      0.929156       68.4565       1.00000       32.5933  0.000000E+00       8.97642  0.000000E+00       8.97642  0.000000E+00  0.000000E+00      -17.9528       76.4175       3.81646        1       Scatag_D
      40       1719.47       5304.80  0.000000E+00  0.000000E+00       2230.42  0.000000E+00       2510.82       6443.62  0.000000E+00       10848.1       52.9735       9807.42 -1.000000E+10 -1.000000E+10 -1.000000E+10 -1.000000E+10 -1.000000E+10  0.000000E+00  0.000000E+00  0.000000E+00  0.000000E+00  0.000000E+00  0.000000E+00  0.000000E+00  0.000000E+00  0.000000E+00  0.000000E+00  0.000000E+00  0.000000E+00       Funtag_D
      41  4.246062E+05  3.856530E+05       38953.2       16.4772     0.0977423      0.637255      0.185043      0.227598     0.0636557   -0.00268916    0.00781508      0.986426       Opttag_D
      41       75.2209       1.13762       1.00000  0.000000E+00       1.00000  0.000000E+00      0.929124       68.5176       1.00000       32.5940  0.000000E+00       8.97328  0.000000E+00       8.97328  0.000000E+00  0.000000E+00      -17.9466       76.4175       3.81646        1       Scatag_D
      41       1715.53       5303.48  0.000000E+00  0.000000E+00       2232.81  0.000000E+00       2514.48       6467.32  0.000000E+00       10861.2       53.3467       9805.00 -1.000000E+10 -1.000000E+10 -1.000000E+10 -1.000000E+10 -1.000000E+10  0.000000E+00  0.000000E+00  0.000000E+00  0.000000E+00  0.000000E+00  0.000000E+00  0.000000E+00  0.000000E+00  0.000000E+00  0.000000E+00  0.000000E+00  0.000000E+00       Funtag_D
      42  4.244845E+05  3.854707E+05       39013.8       16.8648     0.0994580      0.659921      0.184929      0.227552     0.0641701   -0.00270461    0.00780382      0.987354       Opttag_D
      42       75.2713       1.13499       1.00000  0.000000E+00       1.00000  0.000000E+00      0.929081       68.6116       1.00000       32.5958  0.000000E+00       8.96852  0.000000E+00       8.96852  0.000000E+00  0.000000E+00      -17.9370       76.4175       3.81646        1       Scatag_D
      42       1710.59       5313.46  0.000000E+00  0.000000E+00       2236.48  0.000000E+00       2518.63       6503.10  0.000000E+00       10878.6       53.7792       9799.16 -1.000000E+10 -1.000000E+10 -1.000000E+10 -1.000000E+10 -1.000000E+10  0.000000E+00  0.000000E+00  0.000000E+00  0.000000E+00  0.000000E+00  0.000000E+00  0.000000E+00  0.000000E+00  0.000000E+00  0.000000E+00  0.000000E+00  0.000000E+00       Funtag_D
      43  4.243978E+05  3.853325E+05       39065.3       20.6016      0.100976      0.679606      0.184840      0.227525     0.0645600   -0.00277536    0.00779481      0.989027       Opttag_D
      43       75.3152       1.13317       1.00000  0.000000E+00       1.00000  0.000000E+00      0.929047       68.6891       1.00000       32.5993  0.000000E+00       8.96498  0.000000E+00       8.96498  0.000000E+00  0.000000E+00      -17.9300       76.4175       3.81646        1       Scatag_D
      43       1706.64       5331.48  0.000000E+00  0.000000E+00       2239.60  0.000000E+00       2521.55       6525.81  0.000000E+00       10892.3       53.7948       9794.08 -1.000000E+10 -1.000000E+10 -1.000000E+10 -1.000000E+10 -1.000000E+10  0.000000E+00  0.000000E+00  0.000000E+00  0.000000E+00  0.000000E+00  0.000000E+00  0.000000E+00  0.000000E+00  0.000000E+00  0.000000E+00  0.000000E+00  0.000000E+00       Funtag_D
      44  4.243095E+05  3.852388E+05       39070.7       14.6957      0.102141      0.694475      0.184782      0.227514     0.0648247   -0.00288559    0.00778073      0.988489       Opttag_D
      44       75.3480       1.13201       1.00000  0.000000E+00       1.00000  0.000000E+00      0.929026       68.7429       1.00000       32.6024  0.000000E+00       8.96289  0.000000E+00       8.96289  0.000000E+00  0.000000E+00      -17.9258       76.4175       3.81646        1       Scatag_D
      44       1700.48       5325.68  0.000000E+00  0.000000E+00       2242.09  0.000000E+00       2522.51       6535.87  0.000000E+00       10902.0       53.5012       9788.55 -1.000000E+10 -1.000000E+10 -1.000000E+10 -1.000000E+10 -1.000000E+10  0.000000E+00  0.000000E+00  0.000000E+00  0.000000E+00  0.000000E+00  0.000000E+00  0.000000E+00  0.000000E+00  0.000000E+00  0.000000E+00  0.000000E+00  0.000000E+00       Funtag_D
      45  4.242165E+05  3.851203E+05       39096.2       14.6449      0.103772      0.714322      0.184709      0.227493     0.0651591   -0.00301047    0.00777546      0.988829       Opttag_D
      45       75.3917       1.13048       1.00000  0.000000E+00       1.00000  0.000000E+00      0.928994       68.8094       1.00000       32.6094  0.000000E+00       8.96062  0.000000E+00       8.96062  0.000000E+00  0.000000E+00      -17.9212       76.4175       3.81646        1       Scatag_D
      45       1698.18       5329.35  0.000000E+00  0.000000E+00       2245.76  0.000000E+00       2523.15       6547.62  0.000000E+00       10917.2       52.9022       9782.03 -1.000000E+10 -1.000000E+10 -1.000000E+10 -1.000000E+10 -1.000000E+10  0.000000E+00  0.000000E+00  0.000000E+00  0.000000E+00  0.000000E+00  0.000000E+00  0.000000E+00  0.000000E+00  0.000000E+00  0.000000E+00  0.000000E+00  0.000000E+00       Funtag_D
      46  4.241471E+05  3.850193E+05       39127.8       16.6412      0.105171      0.730617      0.184648      0.227457     0.0654439   -0.00305212    0.00777691      0.989401       Opttag_D
      46       75.4274       1.12908       1.00000  0.000000E+00       1.00000  0.000000E+00      0.928967       68.8599       1.00000       32.6184  0.000000E+00       8.95843  0.000000E+00       8.95843  0.000000E+00  0.000000E+00      -17.9169       76.4175       3.81646        1       Scatag_D
      46       1698.82       5335.52  0.000000E+00  0.000000E+00       2248.82  0.000000E+00       2523.55       6560.35  0.000000E+00       10931.2       52.6749       9776.90 -1.000000E+10 -1.000000E+10 -1.000000E+10 -1.000000E+10 -1.000000E+10  0.000000E+00  0.000000E+00  0.000000E+00  0.000000E+00  0.000000E+00  0.000000E+00  0.000000E+00  0.000000E+00  0.000000E+00  0.000000E+00  0.000000E+00  0.000000E+00       Funtag_D
      47  4.240738E+05  3.849203E+05       39153.5       13.7115      0.106526      0.745807      0.184588      0.227418     0.0657234   -0.00305438    0.00777645      0.989627       Opttag_D
      47       75.4607       1.12759       1.00000  0.000000E+00       1.00000  0.000000E+00      0.928942       68.9034       1.00000       32.6300  0.000000E+00       8.95608  0.000000E+00       8.95608  0.000000E+00  0.000000E+00      -17.9122       76.4175       3.81646        1       Scatag_D
      47       1698.62       5337.96  0.000000E+00  0.000000E+00       2251.87  0.000000E+00       2523.99       6570.95  0.000000E+00       10945.8       52.6419       9771.62 -1.000000E+10 -1.000000E+10 -1.000000E+10 -1.000000E+10 -1.000000E+10  0.000000E+00  0.000000E+00  0.000000E+00  0.000000E+00  0.000000E+00  0.000000E+00  0.000000E+00  0.000000E+00  0.000000E+00  0.000000E+00  0.000000E+00  0.000000E+00       Funtag_D
      48  4.239895E+05  3.847775E+05       39212.0       13.8156      0.108474      0.766682      0.184503      0.227355     0.0661265   -0.00303920    0.00778390      0.990257       Opttag_D
      48       75.5072       1.12537       1.00000  0.000000E+00       1.00000  0.000000E+00      0.928907       68.9606       1.00000       32.6527  0.000000E+00       8.95195  0.000000E+00       8.95195  0.000000E+00  0.000000E+00      -17.9039       76.4175       3.81646        1       Scatag_D
      48       1701.87       5344.76  0.000000E+00  0.000000E+00       2256.27  0.000000E+00       2525.86       6599.83  0.000000E+00       10967.2       52.8760       9763.30 -1.000000E+10 -1.000000E+10 -1.000000E+10 -1.000000E+10 -1.000000E+10  0.000000E+00  0.000000E+00  0.000000E+00  0.000000E+00  0.000000E+00  0.000000E+00  0.000000E+00  0.000000E+00  0.000000E+00  0.000000E+00  0.000000E+00  0.000000E+00       Funtag_D
      49  4.239232E+05  3.846475E+05       39275.8       18.3089      0.110460      0.787000      0.184430      0.227319     0.0664934   -0.00304200    0.00779402      0.991628       Opttag_D
      49       75.5535       1.12343       1.00000  0.000000E+00       1.00000  0.000000E+00      0.928876       69.0168       1.00000       32.6809  0.000000E+00       8.94732  0.000000E+00       8.94732  0.000000E+00  0.000000E+00      -17.8946       76.4175       3.81646        1       Scatag_D
      49       1706.30       5359.57  0.000000E+00  0.000000E+00       2260.79  0.000000E+00       2529.06       6623.90  0.000000E+00       10989.9       53.0394       9753.21 -1.000000E+10 -1.000000E+10 -1.000000E+10 -1.000000E+10 -1.000000E+10  0.000000E+00  0.000000E+00  0.000000E+00  0.000000E+00  0.000000E+00  0.000000E+00  0.000000E+00  0.000000E+00  0.000000E+00  0.000000E+00  0.000000E+00  0.000000E+00       Funtag_D
      50  4.238568E+05  3.845763E+05       39280.4       12.6831      0.111541      0.797355      0.184389      0.227302     0.0666943   -0.00307796    0.00779231      0.991738       Opttag_D
      50       75.5775       1.12224       1.00000  0.000000E+00       1.00000  0.000000E+00      0.928863       69.0421       1.00000       32.6995  0.000000E+00       8.94491  0.000000E+00       8.94491  0.000000E+00  0.000000E+00      -17.8898       76.4175       3.81646        1       Scatag_D
      50       1705.55       5360.75  0.000000E+00  0.000000E+00       2263.30  0.000000E+00       2531.05       6619.59  0.000000E+00       11002.0       53.1650       9745.01 -1.000000E+10 -1.000000E+10 -1.000000E+10 -1.000000E+10 -1.000000E+10  0.000000E+00  0.000000E+00  0.000000E+00  0.000000E+00  0.000000E+00  0.000000E+00  0.000000E+00  0.000000E+00  0.000000E+00  0.000000E+00  0.000000E+00  0.000000E+00       Funtag_D
      51  4.237876E+05  3.845007E+05       39287.0       11.2466      0.112864      0.809360      0.184352      0.227302     0.0669077   -0.00313498    0.00779332      0.992113       Opttag_D
      51       75.6059       1.12097       1.00000  0.000000E+00       1.00000  0.000000E+00      0.928849       69.0716       1.00000       32.7232  0.000000E+00       8.94261  0.000000E+00       8.94261  0.000000E+00  0.000000E+00      -17.8852       76.4175       3.81646        1       Scatag_D
      51       1705.99       5364.81  0.000000E+00  0.000000E+00       2266.42  0.000000E+00       2534.00       6613.17  0.000000E+00       11016.2       53.1403       9733.20 -1.000000E+10 -1.000000E+10 -1.000000E+10 -1.000000E+10 -1.000000E+10  0.000000E+00  0.000000E+00  0.000000E+00  0.000000E+00  0.000000E+00  0.000000E+00  0.000000E+00  0.000000E+00  0.000000E+00  0.000000E+00  0.000000E+00  0.000000E+00       Funtag_D
      52  4.237234E+05  3.843994E+05       39324.1       17.5208      0.114939      0.827526      0.184300      0.227325     0.0671936   -0.00324937    0.00780588      0.993115       Opttag_D
      52       75.6494       1.11930       1.00000  0.000000E+00       1.00000  0.000000E+00      0.928828       69.1154       1.00000       32.7612  0.000000E+00       8.93997  0.000000E+00       8.93997  0.000000E+00  0.000000E+00      -17.8799       76.4175       3.81646        1       Scatag_D
      52       1711.49       5375.65  0.000000E+00  0.000000E+00       2271.34  0.000000E+00       2539.19       6615.74  0.000000E+00       11039.9       53.0785       9717.67 -1.000000E+10 -1.000000E+10 -1.000000E+10 -1.000000E+10 -1.000000E+10  0.000000E+00  0.000000E+00  0.000000E+00  0.000000E+00  0.000000E+00  0.000000E+00  0.000000E+00  0.000000E+00  0.000000E+00  0.000000E+00  0.000000E+00  0.000000E+00       Funtag_D
      53  4.236510E+05  3.843209E+05       39330.2       13.8287      0.116727      0.842208      0.184262      0.227351     0.0674152   -0.00335391    0.00781352      0.993288       Opttag_D
      53       75.6853       1.11792       1.00000  0.000000E+00       1.00000  0.000000E+00      0.928814       69.1478       1.00000       32.7962  0.000000E+00       8.93824  0.000000E+00       8.93824  0.000000E+00  0.000000E+00      -17.8765       76.4175       3.81646        1       Scatag_D
      53       1714.85       5377.53  0.000000E+00  0.000000E+00       2275.65  0.000000E+00       2544.22       6600.39  0.000000E+00       11059.7       53.0315       9704.82 -1.000000E+10 -1.000000E+10 -1.000000E+10 -1.000000E+10 -1.000000E+10  0.000000E+00  0.000000E+00  0.000000E+00  0.000000E+00  0.000000E+00  0.000000E+00  0.000000E+00  0.000000E+00  0.000000E+00  0.000000E+00  0.000000E+00  0.000000E+00       Funtag_D
      54  4.235804E+05  3.842532E+05       39327.2       12.3846      0.118277      0.854051      0.184231      0.227379     0.0676063   -0.00346694    0.00782392      0.993372       Opttag_D
      54       75.7153       1.11653       1.00000  0.000000E+00       1.00000  0.000000E+00      0.928802       69.1682       1.00000       32.8297  0.000000E+00       8.93746  0.000000E+00       8.93746  0.000000E+00  0.000000E+00      -17.8749       76.4175       3.81646        1       Scatag_D
      54       1719.41       5378.43  0.000000E+00  0.000000E+00       2279.49  0.000000E+00       2548.45       6579.87  0.000000E+00       11074.4       53.0308       9694.09 -1.000000E+10 -1.000000E+10 -1.000000E+10 -1.000000E+10 -1.000000E+10  0.000000E+00  0.000000E+00  0.000000E+00  0.000000E+00  0.000000E+00  0.000000E+00  0.000000E+00  0.000000E+00  0.000000E+00  0.000000E+00  0.000000E+00  0.000000E+00       Funtag_D
      55  4.235151E+05  3.841589E+05       39356.2       15.5997      0.120488      0.870108      0.184190      0.227429     0.0678721   -0.00366798    0.00784313      0.993715       Opttag_D
      55       75.7568       1.11450       1.00000  0.000000E+00       1.00000  0.000000E+00      0.928787       69.1914       1.00000       32.8819  0.000000E+00       8.93575  0.000000E+00       8.93575  0.000000E+00  0.000000E+00      -17.8715       76.4175       3.81646        1       Scatag_D
      55       1727.87       5382.15  0.000000E+00  0.000000E+00       2285.06  0.000000E+00       2554.34       6576.30  0.000000E+00       11095.0       53.0646       9682.41 -1.000000E+10 -1.000000E+10 -1.000000E+10 -1.000000E+10 -1.000000E+10  0.000000E+00  0.000000E+00  0.000000E+00  0.000000E+00  0.000000E+00  0.000000E+00  0.000000E+00  0.000000E+00  0.000000E+00  0.000000E+00  0.000000E+00  0.000000E+00       Funtag_D
      56  4.234641E+05  3.841101E+05       39353.9       12.3285      0.121664      0.877777      0.184173      0.227457     0.0680099   -0.00379520    0.00785179      0.993795       Opttag_D
      56       75.7778       1.11336       1.00000  0.000000E+00       1.00000  0.000000E+00      0.928776       69.1990       1.00000       32.9123  0.000000E+00       8.93511  0.000000E+00       8.93511  0.000000E+00  0.000000E+00      -17.8702       76.4175       3.81646        1       Scatag_D
      56       1731.69       5383.01  0.000000E+00  0.000000E+00       2288.12  0.000000E+00       2556.67       6562.65  0.000000E+00       11102.9       53.1024       9675.80 -1.000000E+10 -1.000000E+10 -1.000000E+10 -1.000000E+10 -1.000000E+10  0.000000E+00  0.000000E+00  0.000000E+00  0.000000E+00  0.000000E+00  0.000000E+00  0.000000E+00  0.000000E+00  0.000000E+00  0.000000E+00  0.000000E+00  0.000000E+00       Funtag_D
      57  4.234196E+05  3.840860E+05       39333.6       10.1664      0.122183      0.880516      0.184165      0.227472     0.0680780   -0.00387273    0.00784771      0.993559       Opttag_D
      57       75.7863       1.11268       1.00000  0.000000E+00       1.00000  0.000000E+00      0.928770       69.1976       1.00000       32.9279  0.000000E+00       8.93474  0.000000E+00       8.93474  0.000000E+00  0.000000E+00      -17.8695       76.4175       3.81646        1       Scatag_D
      57       1729.89       5380.46  0.000000E+00  0.000000E+00       2289.55  0.000000E+00       2556.37       6550.84  0.000000E+00       11103.1       53.1117       9670.33 -1.000000E+10 -1.000000E+10 -1.000000E+10 -1.000000E+10 -1.000000E+10  0.000000E+00  0.000000E+00  0.000000E+00  0.000000E+00  0.000000E+00  0.000000E+00  0.000000E+00  0.000000E+00  0.000000E+00  0.000000E+00  0.000000E+00  0.000000E+00       Funtag_D
      58  4.233565E+05  3.840415E+05       39315.0       12.5501      0.123567      0.888146      0.184153      0.227512     0.0682035   -0.00402281    0.00784225      0.993698       Opttag_D
      58       75.8090       1.11160       1.00000  0.000000E+00       1.00000  0.000000E+00      0.928751       69.2054       1.00000       32.9657  0.000000E+00       8.93343  0.000000E+00       8.93343  0.000000E+00  0.000000E+00      -17.8669       76.4175       3.81646        1       Scatag_D
      58       1727.48       5381.96  0.000000E+00  0.000000E+00       2293.25  0.000000E+00       2556.24       6535.32  0.000000E+00       11108.1       53.2478       9659.41 -1.000000E+10 -1.000000E+10 -1.000000E+10 -1.000000E+10 -1.000000E+10  0.000000E+00  0.000000E+00  0.000000E+00  0.000000E+00  0.000000E+00  0.000000E+00  0.000000E+00  0.000000E+00  0.000000E+00  0.000000E+00  0.000000E+00  0.000000E+00       Funtag_D
      59  4.233287E+05  3.839875E+05       39341.2       23.5705      0.125995      0.901468      0.184142      0.227580     0.0683561   -0.00429261    0.00784524      0.995019       Opttag_D
      59       75.8486       1.11041       1.00000  0.000000E+00       1.00000  0.000000E+00      0.928715       69.2260       1.00000       33.0300  0.000000E+00       8.93134  0.000000E+00       8.93134  0.000000E+00  0.000000E+00      -17.8627       76.4175       3.81646        1       Scatag_D
      59       1728.80       5396.29  0.000000E+00  0.000000E+00       2299.62  0.000000E+00       2558.40       6533.74  0.000000E+00       11122.1       53.6106       9648.60 -1.000000E+10 -1.000000E+10 -1.000000E+10 -1.000000E+10 -1.000000E+10  0.000000E+00  0.000000E+00  0.000000E+00  0.000000E+00  0.000000E+00  0.000000E+00  0.000000E+00  0.000000E+00  0.000000E+00  0.000000E+00  0.000000E+00  0.000000E+00       Funtag_D
      60  4.232726E+05  3.839896E+05       39283.0       10.0699      0.126022      0.901393      0.184142      0.227583     0.0683501   -0.00426505    0.00783236      0.993875       Opttag_D
      60       75.8485       1.11044       1.00000  0.000000E+00       1.00000  0.000000E+00      0.928712       69.2286       1.00000       33.0294  0.000000E+00       8.93143  0.000000E+00       8.93143  0.000000E+00  0.000000E+00      -17.8629       76.4175       3.81646        1       Scatag_D
      60       1723.13       5383.88  0.000000E+00  0.000000E+00       2299.65  0.000000E+00       2557.55       6496.71  0.000000E+00       11122.3       53.6556       9646.13 -1.000000E+10 -1.000000E+10 -1.000000E+10 -1.000000E+10 -1.000000E+10  0.000000E+00  0.000000E+00  0.000000E+00  0.000000E+00  0.000000E+00  0.000000E+00  0.000000E+00  0.000000E+00  0.000000E+00  0.000000E+00  0.000000E+00  0.000000E+00       Funtag_D
      61  4.232372E+05  3.839790E+05       39258.2       7.89395      0.126664      0.904602      0.184137      0.227605     0.0683800   -0.00430219    0.00782520      0.993636       Opttag_D
      61       75.8584       1.11019       1.00000  0.000000E+00       1.00000  0.000000E+00      0.928700       69.2377       1.00000       33.0442  0.000000E+00       8.93112  0.000000E+00       8.93112  0.000000E+00  0.000000E+00      -17.8622       76.4175       3.81646        1       Scatag_D
      61       1719.98       5381.29  0.000000E+00  0.000000E+00       2301.25  0.000000E+00       2557.59       6475.06  0.000000E+00       11127.3       53.7820       9641.89 -1.000000E+10 -1.000000E+10 -1.000000E+10 -1.000000E+10 -1.000000E+10  0.000000E+00  0.000000E+00  0.000000E+00  0.000000E+00  0.000000E+00  0.000000E+00  0.000000E+00  0.000000E+00  0.000000E+00  0.000000E+00  0.000000E+00  0.000000E+00       Funtag_D
      62  4.232012E+05  3.839536E+05       39247.5       9.38618      0.127914      0.910582      0.184131      0.227649     0.0684515   -0.00440438    0.00782222      0.993499       Opttag_D
      62       75.8776       1.10957       1.00000  0.000000E+00       1.00000  0.000000E+00      0.928677       69.2513       1.00000       33.0757  0.000000E+00       8.93037  0.000000E+00       8.93037  0.000000E+00  0.000000E+00      -17.8607       76.4175       3.81646        1       Scatag_D
      62       1718.67       5379.81  0.000000E+00  0.000000E+00       2304.41  0.000000E+00       2558.69       6459.71  0.000000E+00       11136.2       53.8979       9636.15 -1.000000E+10 -1.000000E+10 -1.000000E+10 -1.000000E+10 -1.000000E+10  0.000000E+00  0.000000E+00  0.000000E+00  0.000000E+00  0.000000E+00  0.000000E+00  0.000000E+00  0.000000E+00  0.000000E+00  0.000000E+00  0.000000E+00  0.000000E+00       Funtag_D
      63  4.231531E+05  3.839036E+05       39249.5       17.8348      0.130052      0.919372      0.184113      0.227734     0.0685926   -0.00461012    0.00782193      0.993704       Opttag_D
      63       75.9098       1.10833       1.00000  0.000000E+00       1.00000  0.000000E+00      0.928634       69.2755       1.00000       33.1292  0.000000E+00       8.92961  0.000000E+00       8.92961  0.000000E+00  0.000000E+00      -17.8592       76.4175       3.81646        1       Scatag_D
      63       1718.54       5382.03  0.000000E+00  0.000000E+00       2309.73  0.000000E+00       2561.61       6446.82  0.000000E+00       11150.7       53.7635       9626.28 -1.000000E+10 -1.000000E+10 -1.000000E+10 -1.000000E+10 -1.000000E+10  0.000000E+00  0.000000E+00  0.000000E+00  0.000000E+00  0.000000E+00  0.000000E+00  0.000000E+00  0.000000E+00  0.000000E+00  0.000000E+00  0.000000E+00  0.000000E+00       Funtag_D
      64  4.231046E+05  3.838649E+05       39239.7       9.77728      0.132293      0.928191      0.184101      0.227814     0.0687019   -0.00479860    0.00782250      0.993032       Opttag_D
      64       75.9435       1.10751       1.00000  0.000000E+00       1.00000  0.000000E+00      0.928585       69.3041       1.00000       33.1868  0.000000E+00       8.92844  0.000000E+00       8.92844  0.000000E+00  0.000000E+00      -17.8569       76.4175       3.81646        1       Scatag_D
      64       1718.79       5374.75  0.000000E+00  0.000000E+00       2315.34  0.000000E+00       2565.73       6424.73  0.000000E+00       11166.6       53.6335       9620.12 -1.000000E+10 -1.000000E+10 -1.000000E+10 -1.000000E+10 -1.000000E+10  0.000000E+00  0.000000E+00  0.000000E+00  0.000000E+00  0.000000E+00  0.000000E+00  0.000000E+00  0.000000E+00  0.000000E+00  0.000000E+00  0.000000E+00  0.000000E+00       Funtag_D
      65  4.230742E+05  3.838514E+05       39222.8       7.24695      0.132496      0.927905      0.184094      0.227816     0.0687401   -0.00482508    0.00781873      0.992860       Opttag_D
      65       75.9463       1.10723       1.00000  0.000000E+00       1.00000  0.000000E+00      0.928575       69.3105       1.00000       33.1892  0.000000E+00       8.92890  0.000000E+00       8.92890  0.000000E+00  0.000000E+00      -17.8578       76.4175       3.81646        1       Scatag_D
      65       1717.14       5372.89  0.000000E+00  0.000000E+00       2315.83  0.000000E+00       2566.26       6415.04  0.000000E+00       11165.9       53.4989       9616.17 -1.000000E+10 -1.000000E+10 -1.000000E+10 -1.000000E+10 -1.000000E+10  0.000000E+00  0.000000E+00  0.000000E+00  0.000000E+00  0.000000E+00  0.000000E+00  0.000000E+00  0.000000E+00  0.000000E+00  0.000000E+00  0.000000E+00  0.000000E+00       Funtag_D
      66  4.230232E+05  3.838123E+05       39210.8       11.0924      0.133245      0.934250      0.184067      0.227828     0.0688504   -0.00492898    0.00781098      0.992880       Opttag_D
      66       75.9565       1.10654       1.00000  0.000000E+00       1.00000  0.000000E+00      0.928541       69.3366       1.00000       33.1973  0.000000E+00       8.93008  0.000000E+00       8.93008  0.000000E+00  0.000000E+00      -17.8602       76.4175       3.81646        1       Scatag_D
      66       1713.73       5373.11  0.000000E+00  0.000000E+00       2317.58  0.000000E+00       2568.47       6413.47  0.000000E+00       11165.9       53.3023       9605.32 -1.000000E+10 -1.000000E+10 -1.000000E+10 -1.000000E+10 -1.000000E+10  0.000000E+00  0.000000E+00  0.000000E+00  0.000000E+00  0.000000E+00  0.000000E+00  0.000000E+00  0.000000E+00  0.000000E+00  0.000000E+00  0.000000E+00  0.000000E+00       Funtag_D
      67  4.230004E+05  3.837703E+05       39230.1       18.1312      0.134556      0.952400      0.184042      0.227868     0.0689689   -0.00508665    0.00780730      0.993387       Opttag_D
      67       75.9747       1.10587       1.00000  0.000000E+00       1.00000  0.000000E+00      0.928489       69.3743       1.00000       33.2176  0.000000E+00       8.93117  0.000000E+00       8.93117  0.000000E+00  0.000000E+00      -17.8623       76.4175       3.81646        1       Scatag_D
      67       1712.12       5378.59  0.000000E+00  0.000000E+00       2320.74  0.000000E+00       2571.82       6425.94  0.000000E+00       11171.9       53.4105       9595.58 -1.000000E+10 -1.000000E+10 -1.000000E+10 -1.000000E+10 -1.000000E+10  0.000000E+00  0.000000E+00  0.000000E+00  0.000000E+00  0.000000E+00  0.000000E+00  0.000000E+00  0.000000E+00  0.000000E+00  0.000000E+00  0.000000E+00  0.000000E+00       Funtag_D
      68  4.229641E+05  3.837606E+05       39203.5       10.2781      0.134702      0.954534      0.184036      0.227876     0.0689963   -0.00512722    0.00780322      0.993319       Opttag_D
      68       75.9762       1.10566       1.00000  0.000000E+00       1.00000  0.000000E+00      0.928480       69.3807       1.00000       33.2174  0.000000E+00       8.93134  0.000000E+00       8.93134  0.000000E+00  0.000000E+00      -17.8627       76.4175       3.81646        1       Scatag_D
      68       1710.33       5377.86  0.000000E+00  0.000000E+00       2321.06  0.000000E+00       2571.83       6402.80  0.000000E+00       11172.4       53.5618       9593.67 -1.000000E+10 -1.000000E+10 -1.000000E+10 -1.000000E+10 -1.000000E+10  0.000000E+00  0.000000E+00  0.000000E+00  0.000000E+00  0.000000E+00  0.000000E+00  0.000000E+00  0.000000E+00  0.000000E+00  0.000000E+00  0.000000E+00  0.000000E+00       Funtag_D
      69  4.229174E+05  3.837211E+05       39196.3       7.99301      0.135601      0.966922      0.184011      0.227916     0.0691079   -0.00529510    0.00779958      0.993899       Opttag_D
      69       75.9874       1.10486       1.00000  0.000000E+00       1.00000  0.000000E+00      0.928439       69.4098       1.00000       33.2268  0.000000E+00       8.93193  0.000000E+00       8.93193  0.000000E+00  0.000000E+00      -17.8639       76.4175       3.81646        1       Scatag_D
      69       1708.74       5384.15  0.000000E+00  0.000000E+00       2323.13  0.000000E+00       2573.19       6388.96  0.000000E+00       11177.3       54.0263       9586.79 -1.000000E+10 -1.000000E+10 -1.000000E+10 -1.000000E+10 -1.000000E+10  0.000000E+00  0.000000E+00  0.000000E+00  0.000000E+00  0.000000E+00  0.000000E+00  0.000000E+00  0.000000E+00  0.000000E+00  0.000000E+00  0.000000E+00  0.000000E+00       Funtag_D
      70  4.228872E+05  3.836746E+05       39212.5       8.96868      0.136561      0.979844      0.183984      0.227959     0.0692390   -0.00546416    0.00780453      0.994631       Opttag_D
      70       75.9992       1.10382       1.00000  0.000000E+00       1.00000  0.000000E+00      0.928396       69.4368       1.00000       33.2396  0.000000E+00       8.93244  0.000000E+00       8.93244  0.000000E+00  0.000000E+00      -17.8649       76.4175       3.81646        1       Scatag_D
      70       1710.91       5392.07  0.000000E+00  0.000000E+00       2325.30  0.000000E+00       2575.29       6391.26  0.000000E+00       11183.1       54.2907       9580.32 -1.000000E+10 -1.000000E+10 -1.000000E+10 -1.000000E+10 -1.000000E+10  0.000000E+00  0.000000E+00  0.000000E+00  0.000000E+00  0.000000E+00  0.000000E+00  0.000000E+00  0.000000E+00  0.000000E+00  0.000000E+00  0.000000E+00  0.000000E+00       Funtag_D
      71  4.228470E+05  3.835336E+05       39313.3       21.6943      0.139000       1.01174      0.183906      0.228075     0.0696369   -0.00588233    0.00784402      0.997856       Opttag_D
      71       76.0277       1.10028       1.00000  0.000000E+00       1.00000  0.000000E+00      0.928283       69.4987       1.00000       33.2743  0.000000E+00       8.93380  0.000000E+00       8.93380  0.000000E+00  0.000000E+00      -17.8676       76.4175       3.81646        1       Scatag_D
      71       1728.26       5427.10  0.000000E+00  0.000000E+00       2330.62  0.000000E+00       2581.23       6427.29  0.000000E+00       11199.5       54.6282       9564.67 -1.000000E+10 -1.000000E+10 -1.000000E+10 -1.000000E+10 -1.000000E+10  0.000000E+00  0.000000E+00  0.000000E+00  0.000000E+00  0.000000E+00  0.000000E+00  0.000000E+00  0.000000E+00  0.000000E+00  0.000000E+00  0.000000E+00  0.000000E+00       Funtag_D
      72  4.227961E+05  3.834651E+05       39330.9       9.61298      0.140272       1.02775      0.183868      0.228123     0.0698303   -0.00605102    0.00785970      0.998152       Opttag_D
      72       76.0424       1.09856       1.00000  0.000000E+00       1.00000  0.000000E+00      0.928223       69.5288       1.00000       33.2963  0.000000E+00       8.93433  0.000000E+00       8.93433  0.000000E+00  0.000000E+00      -17.8687       76.4175       3.81646        1       Scatag_D
      72       1735.18       5430.32  0.000000E+00  0.000000E+00       2333.32  0.000000E+00       2584.78       6427.27  0.000000E+00       11208.5       54.6399       9556.97 -1.000000E+10 -1.000000E+10 -1.000000E+10 -1.000000E+10 -1.000000E+10  0.000000E+00  0.000000E+00  0.000000E+00  0.000000E+00  0.000000E+00  0.000000E+00  0.000000E+00  0.000000E+00  0.000000E+00  0.000000E+00  0.000000E+00  0.000000E+00       Funtag_D
      73  4.227746E+05  3.834538E+05       39320.8       6.73207      0.140099       1.02561      0.183863      0.228108     0.0698623   -0.00599843    0.00786131      0.997957       Opttag_D
      73       76.0398       1.09813       1.00000  0.000000E+00       1.00000  0.000000E+00      0.928228       69.5217       1.00000       33.2932  0.000000E+00       8.93456  0.000000E+00       8.93456  0.000000E+00  0.000000E+00      -17.8691       76.4175       3.81646        1       Scatag_D
      73       1735.89       5428.20  0.000000E+00  0.000000E+00       2332.85  0.000000E+00       2583.84       6423.56  0.000000E+00       11205.6       54.4322       9556.41 -1.000000E+10 -1.000000E+10 -1.000000E+10 -1.000000E+10 -1.000000E+10  0.000000E+00  0.000000E+00  0.000000E+00  0.000000E+00  0.000000E+00  0.000000E+00  0.000000E+00  0.000000E+00  0.000000E+00  0.000000E+00  0.000000E+00  0.000000E+00       Funtag_D
      74  4.227397E+05  3.834023E+05       39337.4       8.11724      0.140345       1.02871      0.183835      0.228100     0.0700076   -0.00595389    0.00787545      0.998235       Opttag_D
      74       76.0411       1.09651       1.00000  0.000000E+00       1.00000  0.000000E+00      0.928208       69.5219       1.00000       33.2976  0.000000E+00       8.93557  0.000000E+00       8.93557  0.000000E+00  0.000000E+00      -17.8711       76.4175       3.81646        1       Scatag_D
      74       1742.14       5431.22  0.000000E+00  0.000000E+00       2333.14  0.000000E+00       2583.49       6437.49  0.000000E+00       11204.2       54.0897       9551.67 -1.000000E+10 -1.000000E+10 -1.000000E+10 -1.000000E+10 -1.000000E+10  0.000000E+00  0.000000E+00  0.000000E+00  0.000000E+00  0.000000E+00  0.000000E+00  0.000000E+00  0.000000E+00  0.000000E+00  0.000000E+00  0.000000E+00  0.000000E+00       Funtag_D
      75  4.227132E+05  3.833214E+05       39391.8       15.8592      0.141414       1.04173      0.183794      0.228110     0.0702359   -0.00601759    0.00789861      0.999378       Opttag_D
      75       76.0508       1.09409       1.00000  0.000000E+00       1.00000  0.000000E+00      0.928147       69.5397       1.00000       33.3185  0.000000E+00       8.93742  0.000000E+00       8.93742  0.000000E+00  0.000000E+00      -17.8748       76.4175       3.81646        1       Scatag_D
      75       1752.40       5443.67  0.000000E+00  0.000000E+00       2335.08  0.000000E+00       2585.22       6471.58  0.000000E+00       11208.0       53.9114       9541.95 -1.000000E+10 -1.000000E+10 -1.000000E+10 -1.000000E+10 -1.000000E+10  0.000000E+00  0.000000E+00  0.000000E+00  0.000000E+00  0.000000E+00  0.000000E+00  0.000000E+00  0.000000E+00  0.000000E+00  0.000000E+00  0.000000E+00  0.000000E+00       Funtag_D
      76  4.226770E+05  3.832862E+05       39390.9       9.29626      0.142128       1.05021      0.183778      0.228125     0.0703353   -0.00607740    0.00790319      0.999830       Opttag_D
      76       76.0575       1.09302       1.00000  0.000000E+00       1.00000  0.000000E+00      0.928108       69.5530       1.00000       33.3337  0.000000E+00       8.93856  0.000000E+00       8.93856  0.000000E+00  0.000000E+00      -17.8771       76.4175       3.81646        1       Scatag_D
      76       1754.43       5448.59  0.000000E+00  0.000000E+00       2336.46  0.000000E+00       2586.39       6463.46  0.000000E+00       11211.5       53.9678       9536.07 -1.000000E+10 -1.000000E+10 -1.000000E+10 -1.000000E+10 -1.000000E+10  0.000000E+00  0.000000E+00  0.000000E+00  0.000000E+00  0.000000E+00  0.000000E+00  0.000000E+00  0.000000E+00  0.000000E+00  0.000000E+00  0.000000E+00  0.000000E+00       Funtag_D
      77  4.226424E+05  3.832445E+05       39397.9       7.29973      0.143348       1.06439      0.183760      0.228154     0.0704529   -0.00621257    0.00790492       1.00066       Opttag_D
      77       76.0687       1.09170       1.00000  0.000000E+00       1.00000  0.000000E+00      0.928043       69.5749       1.00000       33.3620  0.000000E+00       8.94023  0.000000E+00       8.94023  0.000000E+00  0.000000E+00      -17.8805       76.4175       3.81646        1       Scatag_D
      77       1755.20       5457.66  0.000000E+00  0.000000E+00       2338.86  0.000000E+00       2588.23       6459.74  0.000000E+00       11217.8       54.1883       9526.19 -1.000000E+10 -1.000000E+10 -1.000000E+10 -1.000000E+10 -1.000000E+10  0.000000E+00  0.000000E+00  0.000000E+00  0.000000E+00  0.000000E+00  0.000000E+00  0.000000E+00  0.000000E+00  0.000000E+00  0.000000E+00  0.000000E+00  0.000000E+00       Funtag_D
      78  4.226172E+05  3.832147E+05       39402.5       7.92739      0.144268       1.07469      0.183754      0.228174     0.0705369   -0.00630392    0.00790413       1.00115       Opttag_D
      78       76.0758       1.09059       1.00000  0.000000E+00       1.00000  0.000000E+00      0.927989       69.5868       1.00000       33.3865  0.000000E+00       8.94146  0.000000E+00       8.94146  0.000000E+00  0.000000E+00      -17.8829       76.4175       3.81646        1       Scatag_D
      78       1754.85       5462.98  0.000000E+00  0.000000E+00       2340.60  0.000000E+00       2589.54       6461.18  0.000000E+00       11220.8       54.2867       9518.23 -1.000000E+10 -1.000000E+10 -1.000000E+10 -1.000000E+10 -1.000000E+10  0.000000E+00  0.000000E+00  0.000000E+00  0.000000E+00  0.000000E+00  0.000000E+00  0.000000E+00  0.000000E+00  0.000000E+00  0.000000E+00  0.000000E+00  0.000000E+00       Funtag_D
      79  4.225879E+05  3.831739E+05       39414.0       10.9232      0.145985       1.09316      0.183750      0.228208     0.0706521   -0.00649494    0.00790177       1.00202       Opttag_D
      79       76.0868       1.08871       1.00000  0.000000E+00       1.00000  0.000000E+00      0.927882       69.6046       1.00000       33.4365  0.000000E+00       8.94381  0.000000E+00       8.94381  0.000000E+00  0.000000E+00      -17.8876       76.4175       3.81646        1       Scatag_D
      79       1753.80       5472.45  0.000000E+00  0.000000E+00       2343.85  0.000000E+00       2592.02       6467.64  0.000000E+00       11225.1       54.4791       9504.69 -1.000000E+10 -1.000000E+10 -1.000000E+10 -1.000000E+10 -1.000000E+10  0.000000E+00  0.000000E+00  0.000000E+00  0.000000E+00  0.000000E+00  0.000000E+00  0.000000E+00  0.000000E+00  0.000000E+00  0.000000E+00  0.000000E+00  0.000000E+00       Funtag_D
      80  4.225612E+05  3.831724E+05       39388.8       7.13339      0.146212       1.09528      0.183754      0.228204     0.0706565   -0.00645916    0.00789688       1.00161       Opttag_D
      80       76.0859       1.08837       1.00000  0.000000E+00       1.00000  0.000000E+00      0.927857       69.6037       1.00000       33.4449  0.000000E+00       8.94430  0.000000E+00       8.94430  0.000000E+00  0.000000E+00      -17.8886       76.4175       3.81646        1       Scatag_D
      80       1751.63       5467.97  0.000000E+00  0.000000E+00       2344.23  0.000000E+00       2591.93       6456.49  0.000000E+00       11222.0       54.4020       9500.21 -1.000000E+10 -1.000000E+10 -1.000000E+10 -1.000000E+10 -1.000000E+10  0.000000E+00  0.000000E+00  0.000000E+00  0.000000E+00  0.000000E+00  0.000000E+00  0.000000E+00  0.000000E+00  0.000000E+00  0.000000E+00  0.000000E+00  0.000000E+00       Funtag_D
      81  4.225399E+05  3.831784E+05       39361.5       7.73931      0.146202       1.09478      0.183762      0.228191     0.0706394   -0.00638777    0.00789291       1.00105       Opttag_D
      81       76.0826       1.08828       1.00000  0.000000E+00       1.00000  0.000000E+00      0.927842       69.6000       1.00000       33.4464  0.000000E+00       8.94460  0.000000E+00       8.94460  0.000000E+00  0.000000E+00      -17.8892       76.4175       3.81646        1       Scatag_D
      81       1749.87       5461.93  0.000000E+00  0.000000E+00       2344.15  0.000000E+00       2591.25       6446.98  0.000000E+00       11216.9       54.2605       9496.18 -1.000000E+10 -1.000000E+10 -1.000000E+10 -1.000000E+10 -1.000000E+10  0.000000E+00  0.000000E+00  0.000000E+00  0.000000E+00  0.000000E+00  0.000000E+00  0.000000E+00  0.000000E+00  0.000000E+00  0.000000E+00  0.000000E+00  0.000000E+00       Funtag_D
      82  4.225102E+05  3.831848E+05       39325.5       9.42856      0.146600       1.09798      0.183774      0.228178     0.0706215   -0.00630338    0.00789062       1.00013       Opttag_D
      82       76.0792       1.08788       1.00000  0.000000E+00       1.00000  0.000000E+00      0.927793       69.6006       1.00000       33.4587  0.000000E+00       8.94586  0.000000E+00       8.94586  0.000000E+00  0.000000E+00      -17.8917       76.4175       3.81646        1       Scatag_D
      82       1748.86       5451.90  0.000000E+00  0.000000E+00       2344.80  0.000000E+00       2590.75       6438.38  0.000000E+00       11209.5       54.0100       9487.22 -1.000000E+10 -1.000000E+10 -1.000000E+10 -1.000000E+10 -1.000000E+10  0.000000E+00  0.000000E+00  0.000000E+00  0.000000E+00  0.000000E+00  0.000000E+00  0.000000E+00  0.000000E+00  0.000000E+00  0.000000E+00  0.000000E+00  0.000000E+00       Funtag_D
      83  4.224862E+05  3.831965E+05       39289.7       14.0827      0.147529       1.10569      0.183795      0.228170     0.0705883   -0.00622405    0.00789156      0.999170       Opttag_D
      83       76.0772       1.08736       1.00000  0.000000E+00       1.00000  0.000000E+00      0.927708       69.6086       1.00000       33.4835  0.000000E+00       8.94784  0.000000E+00       8.94784  0.000000E+00  0.000000E+00      -17.8957       76.4175       3.81646        1       Scatag_D
      83       1749.27       5441.40  0.000000E+00  0.000000E+00       2346.44  0.000000E+00       2590.53       6433.31  0.000000E+00       11201.5       53.6898       9473.54 -1.000000E+10 -1.000000E+10 -1.000000E+10 -1.000000E+10 -1.000000E+10  0.000000E+00  0.000000E+00  0.000000E+00  0.000000E+00  0.000000E+00  0.000000E+00  0.000000E+00  0.000000E+00  0.000000E+00  0.000000E+00  0.000000E+00  0.000000E+00       Funtag_D
      84  4.224586E+05  3.831938E+05       39264.8       7.22682      0.147868       1.10866      0.183799      0.228179     0.0705958   -0.00626588    0.00788871      0.998945       Opttag_D
      84       76.0786       1.08713       1.00000  0.000000E+00       1.00000  0.000000E+00      0.927688       69.6126       1.00000       33.4912  0.000000E+00       8.94854  0.000000E+00       8.94854  0.000000E+00  0.000000E+00      -17.8971       76.4175       3.81646        1       Scatag_D
      84       1748.01       5438.95  0.000000E+00  0.000000E+00       2347.04  0.000000E+00       2590.02       6416.92  0.000000E+00       11201.1       53.6421       9469.06 -1.000000E+10 -1.000000E+10 -1.000000E+10 -1.000000E+10 -1.000000E+10  0.000000E+00  0.000000E+00  0.000000E+00  0.000000E+00  0.000000E+00  0.000000E+00  0.000000E+00  0.000000E+00  0.000000E+00  0.000000E+00  0.000000E+00  0.000000E+00       Funtag_D
      85  4.224358E+05  3.831890E+05       39246.8       6.45707      0.148524       1.11431      0.183802      0.228200     0.0706096   -0.00634395    0.00788628      0.998744       Opttag_D
      85       76.0813       1.08671       1.00000  0.000000E+00       1.00000  0.000000E+00      0.927650       69.6197       1.00000       33.5063  0.000000E+00       8.94969  0.000000E+00       8.94969  0.000000E+00  0.000000E+00      -17.8994       76.4175       3.81646        1       Scatag_D
      85       1746.93       5436.76  0.000000E+00  0.000000E+00       2348.22  0.000000E+00       2589.00       6411.22  0.000000E+00       11200.5       53.5645       9460.54 -1.000000E+10 -1.000000E+10 -1.000000E+10 -1.000000E+10 -1.000000E+10  0.000000E+00  0.000000E+00  0.000000E+00  0.000000E+00  0.000000E+00  0.000000E+00  0.000000E+00  0.000000E+00  0.000000E+00  0.000000E+00  0.000000E+00  0.000000E+00       Funtag_D
      86  4.224147E+05  3.831877E+05       39226.9       7.24791      0.149292       1.12039      0.183812      0.228224     0.0706132   -0.00641009    0.00788233      0.998388       Opttag_D
      86       76.0832       1.08623       1.00000  0.000000E+00       1.00000  0.000000E+00      0.927602       69.6244       1.00000       33.5253  0.000000E+00       8.95087  0.000000E+00       8.95087  0.000000E+00  0.000000E+00      -17.9017       76.4175       3.81646        1       Scatag_D
      86       1745.18       5432.89  0.000000E+00  0.000000E+00       2349.58  0.000000E+00       2587.64       6411.36  0.000000E+00       11198.0       53.4216       9448.84 -1.000000E+10 -1.000000E+10 -1.000000E+10 -1.000000E+10 -1.000000E+10  0.000000E+00  0.000000E+00  0.000000E+00  0.000000E+00  0.000000E+00  0.000000E+00  0.000000E+00  0.000000E+00  0.000000E+00  0.000000E+00  0.000000E+00  0.000000E+00       Funtag_D
      87  4.223904E+05  3.831909E+05       39199.5       16.0818      0.151238       1.13463      0.183841      0.228303     0.0706043   -0.00660562    0.00787231      0.998015       Opttag_D
      87       76.0880       1.08524       1.00000  0.000000E+00       1.00000  0.000000E+00      0.927484       69.6335       1.00000       33.5742  0.000000E+00       8.95344  0.000000E+00       8.95344  0.000000E+00  0.000000E+00      -17.9069       76.4175       3.81646        1       Scatag_D
      87       1740.75       5428.82  0.000000E+00  0.000000E+00       2353.25  0.000000E+00       2583.97       6430.91  0.000000E+00       11192.1       53.0889       9416.68 -1.000000E+10 -1.000000E+10 -1.000000E+10 -1.000000E+10 -1.000000E+10  0.000000E+00  0.000000E+00  0.000000E+00  0.000000E+00  0.000000E+00  0.000000E+00  0.000000E+00  0.000000E+00  0.000000E+00  0.000000E+00  0.000000E+00  0.000000E+00       Funtag_D
      88  4.223552E+05  3.831940E+05       39161.2       7.25186      0.151765       1.13787      0.183851      0.228319     0.0705954   -0.00660842    0.00786785      0.997464       Opttag_D
      88       76.0884       1.08498       1.00000  0.000000E+00       1.00000  0.000000E+00      0.927451       69.6339       1.00000       33.5873  0.000000E+00       8.95400  0.000000E+00       8.95400  0.000000E+00  0.000000E+00      -17.9080       76.4175       3.81646        1       Scatag_D
      88       1738.78       5422.83  0.000000E+00  0.000000E+00       2354.30  0.000000E+00       2582.76       6415.53  0.000000E+00       11188.8       52.9838       9405.22 -1.000000E+10 -1.000000E+10 -1.000000E+10 -1.000000E+10 -1.000000E+10  0.000000E+00  0.000000E+00  0.000000E+00  0.000000E+00  0.000000E+00  0.000000E+00  0.000000E+00  0.000000E+00  0.000000E+00  0.000000E+00  0.000000E+00  0.000000E+00       Funtag_D
      89  4.223343E+05  3.831929E+05       39141.4       6.75868      0.151968       1.13852      0.183854      0.228324     0.0705986   -0.00657870    0.00786531      0.997237       Opttag_D
      89       76.0878       1.08481       1.00000  0.000000E+00       1.00000  0.000000E+00      0.927437       69.6328       1.00000       33.5905  0.000000E+00       8.95410  0.000000E+00       8.95410  0.000000E+00  0.000000E+00      -17.9082       76.4175       3.81646        1       Scatag_D
      89       1737.66       5420.37  0.000000E+00  0.000000E+00       2354.74  0.000000E+00       2581.59       6411.93  0.000000E+00       11185.2       52.9411       9397.01 -1.000000E+10 -1.000000E+10 -1.000000E+10 -1.000000E+10 -1.000000E+10  0.000000E+00  0.000000E+00  0.000000E+00  0.000000E+00  0.000000E+00  0.000000E+00  0.000000E+00  0.000000E+00  0.000000E+00  0.000000E+00  0.000000E+00  0.000000E+00       Funtag_D
      90  4.223062E+05  3.831935E+05       39112.7       8.18546      0.152719       1.14223      0.183865      0.228349     0.0705968   -0.00656198    0.00786589      0.996887       Opttag_D
      90       76.0882       1.08447       1.00000  0.000000E+00       1.00000  0.000000E+00      0.927392       69.6365       1.00000       33.6040  0.000000E+00       8.95486  0.000000E+00       8.95486  0.000000E+00  0.000000E+00      -17.9097       76.4175       3.81646        1       Scatag_D
      90       1737.91       5416.56  0.000000E+00  0.000000E+00       2356.42  0.000000E+00       2579.66       6414.00  0.000000E+00       11178.8       52.8534       9376.49 -1.000000E+10 -1.000000E+10 -1.000000E+10 -1.000000E+10 -1.000000E+10  0.000000E+00  0.000000E+00  0.000000E+00  0.000000E+00  0.000000E+00  0.000000E+00  0.000000E+00  0.000000E+00  0.000000E+00  0.000000E+00  0.000000E+00  0.000000E+00       Funtag_D
      91  4.222942E+05  3.832023E+05       39091.9       20.4504      0.154328       1.14971      0.183889      0.228400     0.0705720   -0.00654680    0.00787822      0.996554       Opttag_D
      91       76.0902       1.08420       1.00000  0.000000E+00       1.00000  0.000000E+00      0.927301       69.6522       1.00000       33.6296  0.000000E+00       8.95617  0.000000E+00       8.95617  0.000000E+00  0.000000E+00      -17.9123       76.4175       3.81646        1       Scatag_D
      91       1743.37       5412.95  0.000000E+00  0.000000E+00       2360.18  0.000000E+00       2577.64       6442.01  0.000000E+00       11167.8       52.7899       9335.21 -1.000000E+10 -1.000000E+10 -1.000000E+10 -1.000000E+10 -1.000000E+10  0.000000E+00  0.000000E+00  0.000000E+00  0.000000E+00  0.000000E+00  0.000000E+00  0.000000E+00  0.000000E+00  0.000000E+00  0.000000E+00  0.000000E+00  0.000000E+00       Funtag_D
      92  4.222543E+05  3.832011E+05       39053.2       8.17636      0.154539       1.15076      0.183891      0.228410     0.0705755   -0.00657228    0.00787610      0.996236       Opttag_D
      92       76.0908       1.08412       1.00000  0.000000E+00       1.00000  0.000000E+00      0.927292       69.6543       1.00000       33.6333  0.000000E+00       8.95655  0.000000E+00       8.95655  0.000000E+00  0.000000E+00      -17.9131       76.4175       3.81646        1       Scatag_D
      92       1742.43       5409.49  0.000000E+00  0.000000E+00       2360.65  0.000000E+00       2577.59       6413.54  0.000000E+00       11166.9       52.7842       9329.81 -1.000000E+10 -1.000000E+10 -1.000000E+10 -1.000000E+10 -1.000000E+10  0.000000E+00  0.000000E+00  0.000000E+00  0.000000E+00  0.000000E+00  0.000000E+00  0.000000E+00  0.000000E+00  0.000000E+00  0.000000E+00  0.000000E+00  0.000000E+00       Funtag_D
      93  4.222337E+05  3.832007E+05       39033.0       6.17827      0.154943       1.15263      0.183895      0.228426     0.0705765   -0.00661357    0.00787759      0.996061       Opttag_D
      93       76.0920       1.08406       1.00000  0.000000E+00       1.00000  0.000000E+00      0.927273       69.6601       1.00000       33.6391  0.000000E+00       8.95699  0.000000E+00       8.95699  0.000000E+00  0.000000E+00      -17.9140       76.4175       3.81646        1       Scatag_D
      93       1743.09       5407.59  0.000000E+00  0.000000E+00       2361.57  0.000000E+00       2577.55       6406.20  0.000000E+00       11165.0       52.8041       9319.19 -1.000000E+10 -1.000000E+10 -1.000000E+10 -1.000000E+10 -1.000000E+10  0.000000E+00  0.000000E+00  0.000000E+00  0.000000E+00  0.000000E+00  0.000000E+00  0.000000E+00  0.000000E+00  0.000000E+00  0.000000E+00  0.000000E+00  0.000000E+00       Funtag_D
      94  4.222083E+05  3.832037E+05       39004.7       7.83886      0.155879       1.15658      0.183905      0.228460     0.0705682   -0.00669471    0.00788311      0.995804       Opttag_D
      94       76.0946       1.08399       1.00000  0.000000E+00       1.00000  0.000000E+00      0.927228       69.6712       1.00000       33.6537  0.000000E+00       8.95775  0.000000E+00       8.95775  0.000000E+00  0.000000E+00      -17.9155       76.4175       3.81646        1       Scatag_D
      94       1745.53       5404.80  0.000000E+00  0.000000E+00       2363.70  0.000000E+00       2577.43       6405.20  0.000000E+00       11160.1       52.8526       9295.08 -1.000000E+10 -1.000000E+10 -1.000000E+10 -1.000000E+10 -1.000000E+10  0.000000E+00  0.000000E+00  0.000000E+00  0.000000E+00  0.000000E+00  0.000000E+00  0.000000E+00  0.000000E+00  0.000000E+00  0.000000E+00  0.000000E+00  0.000000E+00       Funtag_D
      95  4.221726E+05  3.832129E+05       38959.7       11.4365      0.157285       1.17597      0.183925      0.228520     0.0705420   -0.00687262    0.00787979      0.995512       Opttag_D
      95       76.0992       1.08408       1.00000  0.000000E+00       1.00000  0.000000E+00      0.927162       69.6858       1.00000       33.6787  0.000000E+00       8.95854  0.000000E+00       8.95854  0.000000E+00  0.000000E+00      -17.9171       76.4175       3.81646        1       Scatag_D
      95       1744.06       5401.64  0.000000E+00  0.000000E+00       2366.82  0.000000E+00       2576.83       6407.38  0.000000E+00       11152.3       52.9420       9257.73 -1.000000E+10 -1.000000E+10 -1.000000E+10 -1.000000E+10 -1.000000E+10  0.000000E+00  0.000000E+00  0.000000E+00  0.000000E+00  0.000000E+00  0.000000E+00  0.000000E+00  0.000000E+00  0.000000E+00  0.000000E+00  0.000000E+00  0.000000E+00       Funtag_D
      96  4.221501E+05  3.832420E+05       38908.1       14.0427      0.159571       1.22735      0.183970      0.228603     0.0704600   -0.00712751    0.00787984      0.995095       Opttag_D
      96       76.1068       1.08455       1.00000  0.000000E+00       1.00000  0.000000E+00      0.927058       69.7074       1.00000       33.7236  0.000000E+00       8.95996  0.000000E+00       8.95996  0.000000E+00  0.000000E+00      -17.9199       76.4175       3.81646        1       Scatag_D
      96       1744.08       5397.11  0.000000E+00  0.000000E+00       2372.10  0.000000E+00       2575.81       6417.02  0.000000E+00       11142.3       53.0732       9206.65 -1.000000E+10 -1.000000E+10 -1.000000E+10 -1.000000E+10 -1.000000E+10  0.000000E+00  0.000000E+00  0.000000E+00  0.000000E+00  0.000000E+00  0.000000E+00  0.000000E+00  0.000000E+00  0.000000E+00  0.000000E+00  0.000000E+00  0.000000E+00       Funtag_D
      97  4.221182E+05  3.832409E+05       38877.4       6.23636      0.159232       1.21996      0.183964      0.228595     0.0704631   -0.00710800    0.00786891      0.994782       Opttag_D
      97       76.1061       1.08462       1.00000  0.000000E+00       1.00000  0.000000E+00      0.927071       69.7045       1.00000       33.7176  0.000000E+00       8.95977  0.000000E+00       8.95977  0.000000E+00  0.000000E+00      -17.9195       76.4175       3.81646        1       Scatag_D
      97       1739.25       5393.71  0.000000E+00  0.000000E+00       2371.16  0.000000E+00       2574.15       6397.26  0.000000E+00       11142.2       53.0047       9206.66 -1.000000E+10 -1.000000E+10 -1.000000E+10 -1.000000E+10 -1.000000E+10  0.000000E+00  0.000000E+00  0.000000E+00  0.000000E+00  0.000000E+00  0.000000E+00  0.000000E+00  0.000000E+00  0.000000E+00  0.000000E+00  0.000000E+00  0.000000E+00       Funtag_D
      98  4.220982E+05  3.832465E+05       38851.8       6.36065      0.159191       1.21905      0.183967      0.228601     0.0704474   -0.00711857    0.00785918      0.994599       Opttag_D
      98       76.1065       1.08486       1.00000  0.000000E+00       1.00000  0.000000E+00      0.927071       69.7048       1.00000       33.7179  0.000000E+00       8.96000  0.000000E+00       8.96000  0.000000E+00  0.000000E+00      -17.9200       76.4175       3.81646        1       Scatag_D
      98       1734.95       5391.73  0.000000E+00  0.000000E+00       2370.87  0.000000E+00       2571.64       6389.64  0.000000E+00       11140.9       52.9633       9199.06 -1.000000E+10 -1.000000E+10 -1.000000E+10 -1.000000E+10 -1.000000E+10  0.000000E+00  0.000000E+00  0.000000E+00  0.000000E+00  0.000000E+00  0.000000E+00  0.000000E+00  0.000000E+00  0.000000E+00  0.000000E+00  0.000000E+00  0.000000E+00       Funtag_D
      99  4.220730E+05  3.832654E+05       38807.7       8.41133      0.159700       1.23002      0.183983      0.228633     0.0703941   -0.00720079    0.00784786      0.994331       Opttag_D
      99       76.1089       1.08541       1.00000  0.000000E+00       1.00000  0.000000E+00      0.927047       69.7120       1.00000       33.7295  0.000000E+00       8.96100  0.000000E+00       8.96100  0.000000E+00  0.000000E+00      -17.9220       76.4175       3.81646        1       Scatag_D
      99       1729.96       5388.82  0.000000E+00  0.000000E+00       2371.81  0.000000E+00       2567.51       6382.45  0.000000E+00       11137.6       52.9576       9176.51 -1.000000E+10 -1.000000E+10 -1.000000E+10 -1.000000E+10 -1.000000E+10  0.000000E+00  0.000000E+00  0.000000E+00  0.000000E+00  0.000000E+00  0.000000E+00  0.000000E+00  0.000000E+00  0.000000E+00  0.000000E+00  0.000000E+00  0.000000E+00       Funtag_D

   MaxLik Scaling Cycle 0105

   Wilson plot

   Statistics in resolution bins after refinement cycle 0003
```


|  |  |
| --- | --- |
|  | - Rxpct factors plot- Rxpct factors table- <Log-Lik Gain>- Ln Sigma A- Correlation Coefficients |


```
   Iters     Tot_Funct    Xray_Funct    Geom_Funct          Grms     rmsD2init     maxD2init             R         Rfree           LLG       LLGfree     rms(bond)    rms(angle)
   Iters             K   B_IMPF_FRAG        K_MISS   B_TEMP_MISS   K_IMPF_MISS   B_IMPF_MISS        K_SOLV   B_TEMP_SOLV   K_IMPF_SOLV   B_IMPF_SOLV        B_TEMP     B_TEMP_11     B_TEMP_12     B_TEMP_22     B_TEMP_13     B_TEMP_23     B_TEMP_33      K_WILSON      B_WILSON      FGCYCLES
   Iters          BOND         ANGLE      IMPROPER       TORSION    SINTORSION      TRIGONAL        PLANAR  BCORRELATION       CONTACT     IDEALDIST        CHIRAL    SIMILARITY       OCCSSUM  UTILDISTANCE     UTILANGLE   UTILTORSION        RHOFIT        ADP-01        ADP-02        ADP-03        ADP-04        ADP-05        ADP-06        ADP-07        ADP-08        ADP-09        ADP-10      RHOFITPC            QM
     100  4.220537E+05  3.833090E+05       38744.7       17.2744      0.160999       1.25715      0.184022      0.228711     0.0702708   -0.00741331    0.00783082      0.994075       Opttag_D
     100       76.1146       1.08665       1.00000  0.000000E+00       1.00000  0.000000E+00      0.926993       69.7292       1.00000       33.7574  0.000000E+00       8.96362  0.000000E+00       8.96362  0.000000E+00  0.000000E+00      -17.9272       76.4175       3.81646        1       Scatag_D
     100       1722.45       5386.05  0.000000E+00  0.000000E+00       2374.36  0.000000E+00       2560.24       6379.41  0.000000E+00       11132.3       53.0023       9136.88 -1.000000E+10 -1.000000E+10 -1.000000E+10 -1.000000E+10 -1.000000E+10  0.000000E+00  0.000000E+00  0.000000E+00  0.000000E+00  0.000000E+00  0.000000E+00  0.000000E+00  0.000000E+00  0.000000E+00  0.000000E+00  0.000000E+00  0.000000E+00       Funtag_D
   Geometry Restraint Deviations SCREEN Information

 
 No -bcorrel_zeroocchydrogens_factor flag found - keeping default: will set B for OCC=0 hydrogens to    1.200 times heavy-atom B


 TLS information. Total number of groups:     2


 Group   Natoms    FixRB   FixTLS   Group Tag    Group Spec
     1     2770      Y        Y     tls1    { A|* }
     2     2770      Y        Y     tls2    { B|* }
 
NOTE BUSTER_TLS_SET tls1 { A|* }
NOTE BUSTER_TLS_T   tls1     -1.00387E-01 -1.94646E-02 -1.04862E-01 -2.75119E-02  4.61084E-02  5.22733E-03
NOTE BUSTER_TLS_L   tls1      1.43977E+00  1.66678E+00  8.28191E-01  2.91853E-01 -4.93436E-01  3.60273E-01
NOTE BUSTER_TLS_S   tls1      2.45347E-01 -2.02592E-01  2.44710E-01 -1.91009E-01 -7.07893E-02 -2.32867E-01 -5.33582E-02 -7.54950E-02
NOTE BUSTER_TLS_O   tls1     -2.34016E+01 -8.37990E+01  1.17779E+01
NOTE BUSTER_TLS_FIX tls1 ALL
 
NOTE BUSTER_TLS_SET tls2 { B|* }
NOTE BUSTER_TLS_T   tls2     -1.34545E-01 -7.70270E-02  4.06613E-02  2.21513E-02  4.79797E-02 -1.95693E-02
NOTE BUSTER_TLS_L   tls2      1.12658E+00  1.16836E+00  9.36760E-01  5.85777E-01 -6.91255E-01 -5.92143E-01
NOTE BUSTER_TLS_S   tls2     -4.81861E-02  5.31306E-02 -8.29736E-02  4.54205E-02  2.18779E-01  5.84247E-02 -8.79871E-02  4.73445E-02
NOTE BUSTER_TLS_O   tls2      2.21707E+01 -7.20650E+01  3.37962E+00
NOTE BUSTER_TLS_FIX tls2 ALL

 For final position, Geometry function summary
     term                           number     weight       rms        GooF      function     fn/numb
     ================================================================================================
     BOND bond lengths (angs)       5655       2.000      0.0078       0.390      1722.45       0.305
     ANGL bond angles (degs)        7674       2.000        0.99       0.592      5386.05       0.702
     TORS split-harmonic tors       1270       0.000       17.60       1.107         0.00       0.000
     SINTOR sinusoidal tors         1270       2.000       17.60                  2374.36       1.870
     PLAN general plane (angs)       963       5.000      0.0146       0.729      2560.24       2.659
     BCORrelations (angs^2)         5655      10.000       0.941       0.336      6379.41       1.128
     CONT (bad contacts)               0       5.000                                 0.00
     IDEAL(ideal-dist contact)      5388       4.000                             11132.30
     CHIRAL (gelly semiharmon)       740       5.000                                53.00
     SIM similarity restraint for NCS and/or TARGET                               9136.88
     OCCSUM restraint on sum of occupanies                                           0.00       as none defined
     DISTAN utility restraints interatomic distance                                  0.00       as none defined
     UTILANGLE utility angle restraints                                              0.00       as none defined
     UTILTOR utility restraints on torsion angles                                    0.00       as none defined
     Peptide omega torsion angles                           2.96 degs - monitor only

 Weighted rms      0.0078         0.994  (temporary rms bond and angle for BUSTER to pick up!)
 Weighted intra-residual rms(bond) for StdProtein (excluding H)      0.0076
 Weighted intra-residual rms(bond) for Buffer     (excluding H)      0.0032
 Outputing geometry progress graph in plotmtv format to file:Geom_Res.mtv

 ############### Running final geometry/screen analysis (screen_final.txt)

 s/r gelly normal termination
```

Statistics and scale factors vs. cycle number
```

|  |  |  |
| --- | --- | --- |
|  | - Rxpct factors- <Log-Lik Gain>- GEOM Residuals- Ln Sigma_A_ | - Scale Factors- B Factors- Imperfection Factors |

```
   Final Geometry Information


---


### 2. Final results

Statistics and scale factors vs. cycle number
```

|  |  |  |
| --- | --- | --- |
|  | - Rxpct factors- <Log-Lik Gain>- GEOM Residuals- Ln Sigma_A_ | - Scale Factors- B Factors- Imperfection Factors |

```
   Average Figure of Merit
   Structure Factor Amplitudes


 # normal termination

  Program stopped.
```

closesok message: time spent in tree = 1263.25 (user) 40.64 (sys)

---

# Normal termination

```
 Resources usage summary:
     Time spent in client ...................  1263.27 (user)       40.66 (system)
     Time spent in scripts (    27 calls) ...     1.64 (user)        0.12 (system)
     Time spent in server ...................     0.00 (user)        0.00 (system)

     Max resident memory used (kb) .......... 445764
     Number of page faults .................. 0 (major)    477950 (minor)
     Number of context switches ............. 7505 (niced)    4744 (other)
```
